# *Mariner* Transposons Contain a Silencer: Possible Role of the Polycomb Repressive Complex 2

**DOI:** 10.1371/journal.pgen.1005902

**Published:** 2016-03-03

**Authors:** Solenne Bire, Sophie Casteret, Benoît Piégu, Linda Beauclair, Nathalie Moiré, Peter Arensbuger, Yves Bigot

**Affiliations:** 1 PRC, UMR INRA-CNRS 7247, PRC, Nouzilly, France; 2 Institute of Biotechnology, University of Lausanne, and Center for Biotechnology UNIL-EPFL, Lausanne, Switzerland; 3 UMR INRA 1282, ISP, Parc de Grandmont, Tours, France; 4 Biological Sciences Department, California State Polytechnic University, Pomona, California, United States of America; University of Utah School of Medicine, UNITED STATES

## Abstract

Transposable elements are driving forces for establishing genetic innovations such as transcriptional regulatory networks in eukaryotic genomes. Here, we describe a silencer situated in the last 300 bp of the *Mos1* transposase open reading frame (ORF) which functions in vertebrate and arthropod cells. Functional silencers are also found at similar locations within three other animal *mariner* elements, i.e. *IS630-Tc1-mariner* (*ITm*) DD34D elements, *Himar1*, *Hsmar1* and *Mcmar1*. These silencers are able to impact eukaryotic promoters monitoring strong, moderate or low expression as well as those of *mariner* elements located upstream of the transposase ORF. We report that the silencing involves at least two transcription factors (TFs) that are conserved within animal species, NFAT-5 and Alx1. These cooperatively act with YY1 to trigger the silencing activity. Four other housekeeping transcription factors (TFs), neuron restrictive silencer factor (NRSF), GAGA factor (GAF) and GTGT factor (GTF), were also found to have binding sites within *mariner* silencers but their impact in modulating the silencer activity remains to be further specified. Interestingly, an NRSF binding site was found to overlap a 30 bp motif coding a highly conserved PHxxYSPDLAPxD peptide in *mariner* transposases. We also present experimental evidence that silencing is mainly achieved by co-opting the host Polycomb Repressive Complex 2 pathway. However, we observe that when PRC2 is impaired another host silencing pathway potentially takes over to maintain weak silencer activity. *Mariner* silencers harbour features of Polycomb Response Elements, which are probably a way for *mariner* elements to self-repress their transcription and mobility in somatic and germinal cells when the required TFs are expressed. At the evolutionary scale, *mariner* elements, through their exaptation, might have been a source of silencers playing a role in the chromatin configuration in eukaryotic genomes.

## Author Summary

Transposons are mobile DNA sequences that have long co-evolved with the genome of their hosts. Consequently, they are involved in the generation of mutations, as well as the creation of genes and regulatory networks. Controlling the transposon activity, and consequently its negative effects on both the host soma and germ line, is a challenge for the survival of both the host and the transposon. To silence transposons, hosts often use defence mechanisms involving DNA methylation and RNA interference pathways. Here we show that *mariner* transposons can self-regulate their activity by using a silencer element located in their DNA sequence. The silencer element interferes with host housekeeping protein transcription factors involved in the polycomb silencing pathways. As the regulation of chromatin configuration by polycomb is an important regulator of animal development, our findings open the possibility that *mariner* silencers might have been exapted during animal evolution to participate in certain regulation pathways of their hosts. Since some of the TFs involved in *mariner* silencer activity play a role at different stages of nervous system development and neuron differentiation, it might be possible that *mariner* transposons can be active during some steps of cell differentiation. Interestingly, *mariner* transposons (i.e. *IS630-Tc1-mariner* (*ITm*) DD34D transposons) have so far only been found in genomes of animals having a nervous system.

## Introduction

Almost all eukaryotic genomes contain transposable elements (TEs). Some of these, known as DNA transposons, move by a simple ‘cut-and-paste’ mechanism removing DNA from one site and inserting it into a new target site. Others, called retrotransposons, move via an RNA intermediate that is copied into DNA and integrated into the genome. The overall fraction of TEs that make up currently described genomes remains difficult to estimate due to the accumulation of several layers of such elements. These layers originate from TE amplification bursts at different periods during the evolution of the element, followed by ageing of the DNA sequence. Recent improvements in sequence analysis methods have showed that the human genome likely consists of at least 66–69% of repeated or repeat-derived sequences [1], which is much higher than the 45–50% that had been reported when this genome was first sequenced. This suggests that the extent to which genomes have been shaped by TEs has probably been underestimated for many eukaryotic species. Mobility, distribution and exaptation of certain TE sequences have been considered as important sources for expansion and diversification of transcriptional regulatory networks as well as for genetic innovations [2,3]. Today, DNA segments derived from TEs that were exapted or inactivated over time by accumulation of mutations appear as remnants of repeated sequences of various ages. While they are rare, active TEs are still present in the genome of extant species in which *de novo* insertions can generate genetic variations. In multicellular eukaryotes TE insertions must occur within the germinal lineage or during early development in order to be transmitted to the following generations. This leads to the suggestion that transposition into somatic cells had no value for the TEs or their host. However, in the early 1980’s evidence began to accumulate showing that somatic TE activity (*i*.*e*. single excision or excision followed by re-insertion) occurred at high frequency in animal taxa. This was first shown for a DNA transposon, *Tc1* in the worm *Caenorhabditis elegans* [4]. Recently, somatic activity was also observed for mammalian LINE-1 and dipteran R2 retrotransposons [5,6]. Interestingly, all of these somatic transpositions occurred in primordial cells associated with neuron-related lineages during embryonic or metamorphic development.

Activation of TE transcription within some cell lineages requires that the factors silencing their expression be specifically switched off in these lineages. The Neuron-Restrictive Silencer Factor (NRSF) that corresponds to the Charlatan (Chn) protein in arthropods [7] and to the SPR-3/SPR-4 in nematodes [8], represses transcription of many neuronal genes in non-neuronal cell types and in neuronal stem cells prior to their differentiation. NRSF binds to a 21 to 30 bp long element called the Neuron-Restrictive Silencer Element [9] (NRSE). NRSF has never been shown before to interfere with TE transcription, even though NRSEs were found in human retrotransposons such as LINE2 [9,10] and that transcription of *Tc1*-like DNA transposons was shown to be activated during development of the *Xenopus* nervous system [11]. We report the existence of a silencer element located in the last 300 bp of the *Mos1* transposase (MOS1) ORF that is functional in both vertebrate and arthropod cells. This silencer is able to interfere with the transposon promoter as well as with promoters of genes located downstream of the silencer sequence. We show that the presence and location of this silencer element is conserved in *mariner*-like elements (MLEs), even though their DNA sequences have significantly diverged. Our data reveal that YY1, NFAT-5, NRSF, Alx1, GAF and GTF proteins have binding sites within these silencer elements. Furthermore, our results are consistent with the hypothesis that these silencers function with the Polycomb Repressive Complexes (PRC). Together, *mariner* silencers might not only regulate the transcription of active MLEs, but might also modify the expression pattern of genes in which active or remnant MLEs are inserted.

## Results

### Characterization of a silencer element in *Mos1*

**Fig 1:**
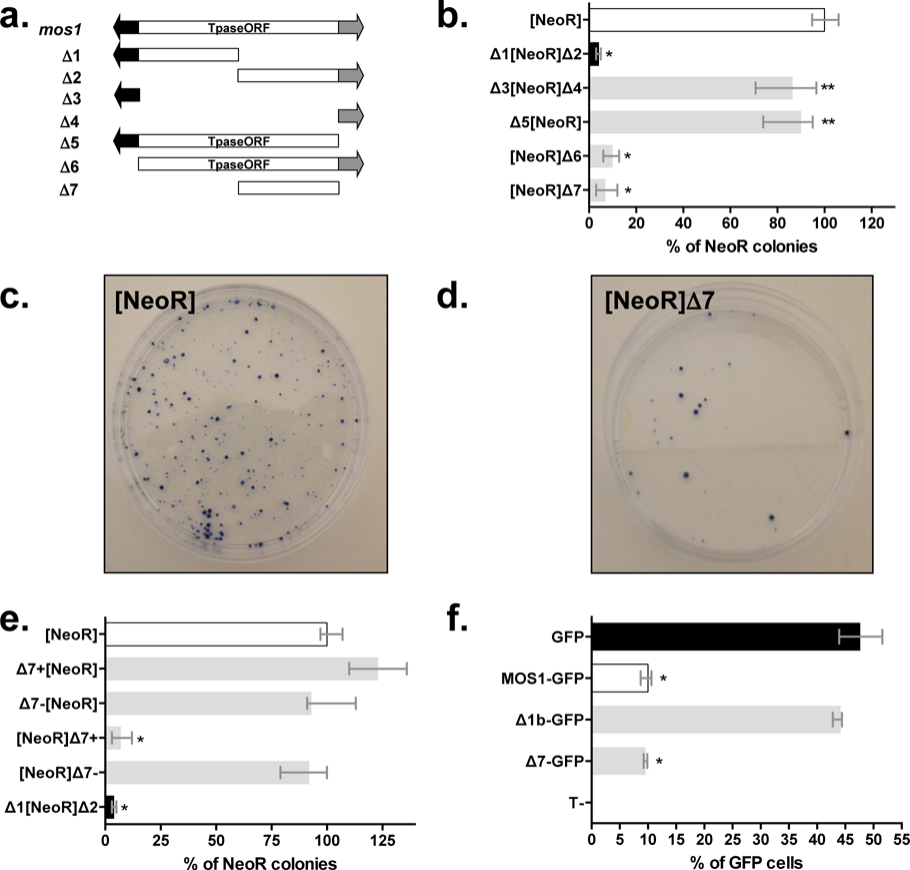
Characterization of a *Mos1* region that negatively interferes with the expression of a marker gene. (**a**) *Mos1* transposon and the various deletion derivatives used herein (Δ1 to Δ7). Blank rectangle: *Mos1* transposase Open Reading Frame (Tpase ORF). Black arrow: 5’ ITR (Inverted Terminal Repeat) fused to the 5’ UTR (UnTranslated Region). Grey arrow: 3’ UTR fused to the 3’ ITR. Δ1 spanned from positions 1 to 655, Δ2 from 656 to 1289, Δ3 from 1 to 174, Δ4 from 1213 to 1289, Δ5 from 1 to 1212, Δ6 from 175 to 1289, and Δ7 from 681 to 1212 of the *Mos1* sequence (Acc N°X78906; 1289 bp). (**b**), (**c**), (**d**), and (**e**) Stable expression assays using the neomycin resistance gene marker in HeLa cells. Different constructs composed of the *NeoR* marker flanked with *Mos1* segments presented in (**a**) at one or both ends were transfected in HeLa cells. Stable integrants were obtained following 15 days of antibiotic selection and resistant colonies were counted. (**b**) Characterization of the segment in the *Mos1* transposon able to silence the expression of the neomycin resistance gene. (**c**) and (**d**) Stained stable integrants obtained after transfection with the control [*NeoR*] construct (~ 275–300 clones) and the [*NeoR*]Δ7 construct (~ 25–30 clones). (**e**) Effects of the location and orientation of the Δ7 segment on the marker gene expression. The Δ7 segment was placed upstream or downstream of the marker gene in the positive (+) or negative (-) orientation. (**f**) Impact of the Δ7-*MOS1* segment intragenic location using a transient gene expression assay. Δ7 and Δ1b were fused in frame to the 5’ end of the *GFP* gene in pCS2+ expression plasmids (pCMV promoter) as previously described [12]. Segment Δ1b (= Δ1 without the 5’ITR-UTR region) or the full length *MOS1* ORF were used as controls. Each construct was transfected into HeLa cells and GFP expression was analysed by fluorescent flow cytometry and compared to HeLa cells transfected with a pCS2-GFP plasmid. Median values from three experiments performed in triplicate are shown. Bars correspond to quartiles 1 and 3. The median value obtain with the control [*NeoR*] construct was fixed at 100% and used as a reference to calculate the medians of the other constructs. Results obtained with [*NeoR*] (white bar) and Δ1[*NeoR*] Δ2 (black bar) were used as benchmarks for all our stable expression assays. * indicates significant difference (p<0.05) compared to the [*NeoR*] or *GFP* control.

Although it was originally used for another purpose (negative controls of transposition done in absence of a transposase source), a stable expression assay was used to investigate whether *Mos1* was able to interfere with the expression of neighbouring genes. This assay consisted in transfecting HeLa cells with plasmids containing a Neomycin Resistance (*NeoR*) marker gene and one or two *Mos1* DNA segments cloned upstream or downstream of the *NeoR* gene (Fig 1A). After two weeks of selection with G418, resistant colonies were stained and counted. The first evidence that *Mos1* could decrease the expression of a marker gene located within its neighbourhood was obtained with the Δ1[*NeoR*]Δ2 construct which corresponded to a complete *Mos1* element containing the *NeoR* gene inserted in its middle. Colony numbers were at least 20-fold lower with the Δ1[*NeoR*]Δ2 construct than with those obtained with the [*NeoR*] reference (Fig 1B). Further constructs were tested in an effort to locate the region responsible for the observed decrease in marker expression within *Mos1*, a region we refer to hereafter as the silencer element. Results obtained with the Δ3[*NeoR*]Δ4 and Δ5[*NeoR*] constructs were not different from those of [*NeoR*] (Fig 1B).

These observations supported two explanations: i) the silencer element was not in the non-coding terminal regions of *Mos1*, ii) the optimal activity of the silencer element was position-dependent and had an effect only when located downstream of the marker. The first explanation was supported by observations based on the [*NeoR*]Δ6 and [*NeoR*]Δ7 constructs which gave results similar to those obtained with the Δ1[*NeoR*]Δ2, indicating that the silencer was located within the 3’ half of the *MOS1* open reading frame (ORF) corresponding to the Δ7 DNA segment (Fig 1B through 1D). The role of the position and orientation of the *Mos1* silencer element was confirmed using four constructs in which the Δ7 DNA segment was cloned upstream or downstream of the *NeoR* gene, in positive (*i*.*e*. with the piece of *MOS1* ORF on the same strand as the *NeoR* ORF) or negative orientations (Fig 1E). Only the [*NeoR*]Δ7+ construct showed a strong silencer effect. Hence, the Δ7 DNA segment had a silencer effect only when located downstream of the marker gene, in the positive orientation with respect to the *NeoR* marker. In addition, complementary experiments demonstrated that an intragenic Δ7 DNA segment in frame with a marker gene had a silencer effect on its expression since Δ7-*GFP* and *MOS1-GFP* fusions expression is similar and significantly lower (~5-fold) than the GFP control (Fig 1F). These data support that Δ7-*MOS1* segment has a silencer effect when it is fused in frame within a gene and that the silencer effect could be operating with the pCMV promoter. These results were confirmed by RT-qPCR using total RNA extracted from transiently transfected cells and *GFP* specific primers.

**Fig 2:**
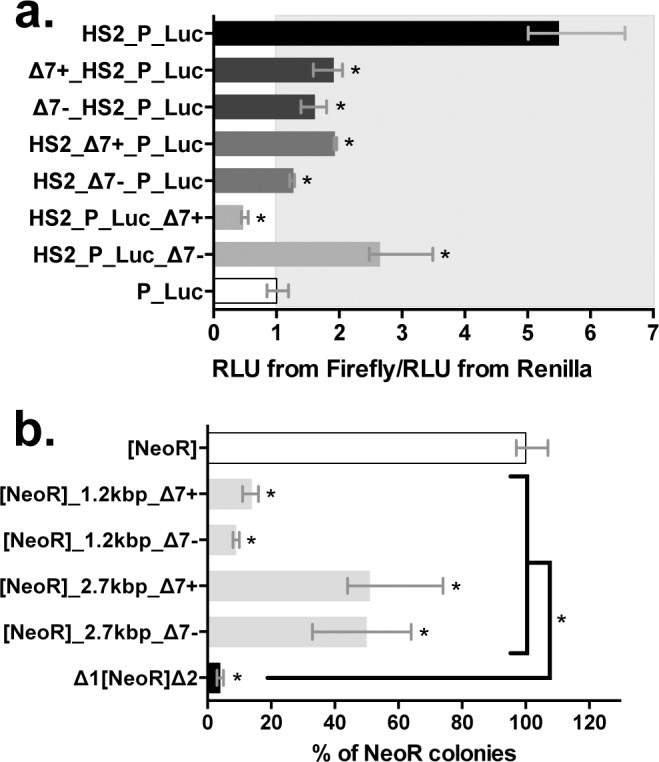
Validation of the silencing feature of the Δ7-*MOS1* segment. (**a**) Effect of Δ7-*MOS1* segment on transient luciferase expression assay in HeLa cells. Twenty-four hours post-transfection, the expression of the *Firefly* and *Renilla* luciferases were measured in a 96-well plate format using the dual luciferase “Stop and Glo” procedure (Promega) and a Berthold plate-reader luminometer. The average expression level from three replicate transfections was normalized to the *Renilla* luciferase co-transfection control. This value was also normalized to the average expression level of the P_Luc plasmid to yield a “fold” enhancement measurement. The ratio “RLU from *Firefly*/RLU from *Renilla*” for the P_Luc transfection is used as a reference and fixed at 1 arbitrary unit. All other ratios were calculated taking this reference into account. Depending on its location in the construct (i.e. Δ7_HS2_P_Luc, HS2_Δ7_P_Luc and HS2_P_Luc_Δ7), an enhancer blocker or silencer function was assigned when the ratio is lower or equal to 1 [14]. Median values from three experiments performed in triplicate are shown. Bars correspond to quartiles 1 and 3. The area where the ratios “RLU from *Firefly*/RLU from *Renilla*” were above 1 (i.e. where no strong silencer effect is observed) is coloured in grey. * indicates a significant difference (p<0.05) with the P_Luc controls. (**b**) Impact of the distance between the Δ7 segment and the marker gene on the transgene expression. The Δ7 segment was located downstream of the marker gene at a distance of 1.2 or 2.7 kbp (the spacers used are DNA fragments cloned from a lambda phage). Each histogram bar corresponds to the median value obtained from three experiments done in triplicate. Bars correspond to quartiles 1 and 3. The median value obtained with the control [*NeoR*] construct was fixed at 100% and used as a reference to calculate the medians of the other constructs. Results obtained with [*NeoR*] (white bar) and Δ1[*NeoR*]Δ2 (black bar) were used as benchmarks for all our stable expression assays. * indicates a significant difference (p<0.05) compared to the [*NeoR*] control.

To confirm that the Δ7-*MOS1* segment contains a real silencer element, we used a transient luciferase expression assay that was previously validated to characterize silencer elements [12] (S1 Fig). Our first results were confirmed with this alternative approach since the only ratio lower than 1 was obtained with the HS2_P_Luc_Δ7+ plasmid (Fig 2A). In addition, they revealed that the expressions of the marker gene in the [*NeoR*]Δ7+ and HS2_P_Luc_Δ7+ constructs are of the same order of magnitude with respect to controls, [*NeoR*] (14.3x; Fig 1B and 1E) and HS2_P_Luc (11.7x; Fig 2A), respectively. Therefore, these data confirmed that the Δ7-*MOS1* segment contained a silencer element which is more efficient when it is located downstream of the maker gene in a positive orientation. They also confirmed that the results obtained with our stable expression assay did not reflect an ability of the plasmid to be integrated into the genome, but the capacity of the *NeoR* gene to be expressed post-integration. Given the above observations we decided that rather than continuing with the transient assay we should use our stable expression assay to investigate the impact of the distance separating the marker and the Δ7 DNA segment by cloning a 1.2 or 2.7 kbp spacer between them. We observed that the 1.2 kbp spacer had little or no impact on Δ7 silencing activity while the 2.7 kbp spacer decreased its activity approximately 5-fold (Fig 2B). It is interesting to notice that a Δ7 DNA segment in the negative orientation located a few kbps away from the marker gene had silencing activity comparable to the one on the Δ7 DNA segment in the positive orientation. These results were verified using linearized constructs (S2 Fig) and were not uniform, suggesting that vector configuration is important in such experiments. An orientation effect similar to that previously observed in the absence of a spacer was found with linearized vectors containing a 3 kbp spacer.

### The *Mos1* silencer element is active in distant species

**Fig 3:**
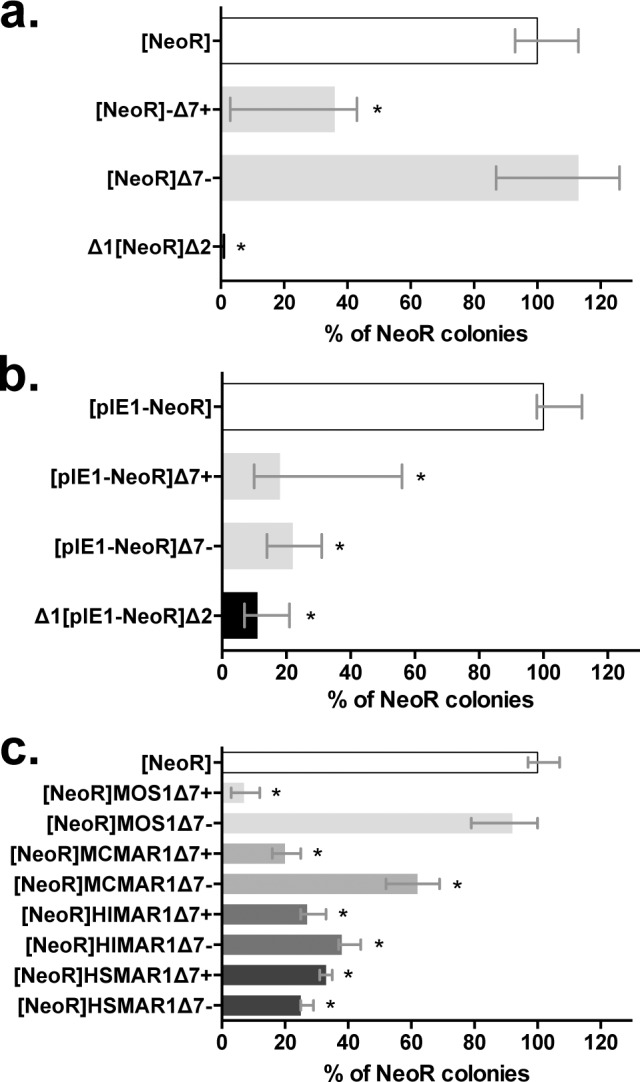
Ubiquitous functioning of Δ7-*MOS1* segment and Δ7 segments originating from 3 other distantly related *mariner* elements in animal cells. Stable expression assays were performed in (**a**) amphibian Speedy cells [13] and (**b**) Insect (Lepidoptera) Sf21 cells. The average number of colonies obtained with the control [*NeoR*] construct in **a**, and [pIE1-*NeoR*] in **b**, were about 50 and 1100, respectively. (**c**) Comparison of orthologous Δ7 segments from *Mos1* (light grey bars), *Mcmar1* (mid-grey bars), *Himar1* (dark grey bars) and *Hsmar1* (black bars) cloned downstream of the marker gene in positive or negative orientations in HeLa cells. Each histogram bar corresponds to the median value obtained from three experiments done in triplicate. Bars correspond to quartiles 1 and 3. The [*NeoR*] construct serves as positive reference and was set to 100%. The Δ1[*NeoR*]Δ2 construct serves as the negative control. * indicates a significant difference (p<0.05) compared to the [*NeoR*] control.

The activity of the *Mos1* silencer element was tested using [*NeoR*]Δ7+ and [*NeoR*]Δ7-, our stable expression assay and two other cellular lineages originating from distantly related species: Speedy cells [13] from *Xenopus tropicalis* (*Amphibia*) and Sf21 cells from *Spodoptera frugiperda* (*Insecta*). Our results showed that the Δ7 DNA segment had a silencer effect in both cellular systems (Fig 3A and 3B), suggesting that the protein factors with which it interferes are conserved in these two species. Interestingly, the orientation effect of the silencer was recovered in Speedy cells but was absent in Sf21 cells.

### The silencer element is conserved among MLEs

The *mariner* TE family consists of five sub-families designated *cecropia*, *elegans/briggsae*, *irritans*, *mauritiana* and *mellifera/capitata* [14]. Based on the phylogeny of its transposase *Mos1* belongs to the *mauritiana* sub-family. The presence of a silencer element was surveyed within the Δ7 DNA segments of three MLEs, *Himar1*, *Mcmar1* and *Hsmar1*, which respectively belong to the *irritans*, *elegans/briggsae*, and *cecropia* sub-families. Results obtained using the stable expression assay (Fig 3C) showed that the Δ7-*MCMAR1* segment had a silencing activity with features similar to those of Δ7-*MOS1* (*i*.*e*. in terms of intensity and orientation). The Δ7-*HIMAR1* and Δ7-*HSMAR1* segments also had silencing activity that was not significantly different from those of Δ7-*MOS1* and Δ7-*MCMAR1*, but independent of their orientation. This result is important because it suggests that the presence of a silencer within the Δ7 DNA segment is a characteristic shared by all MLEs. It also suggests that protein factors conserved in most animal species that interfere with the *mariner* silencer elements might have conserved binding site motifs in their sequences.

### The *Mos1* and *Hsmar1* silencers are able to interfere with their own promoters

**Fig 4:**
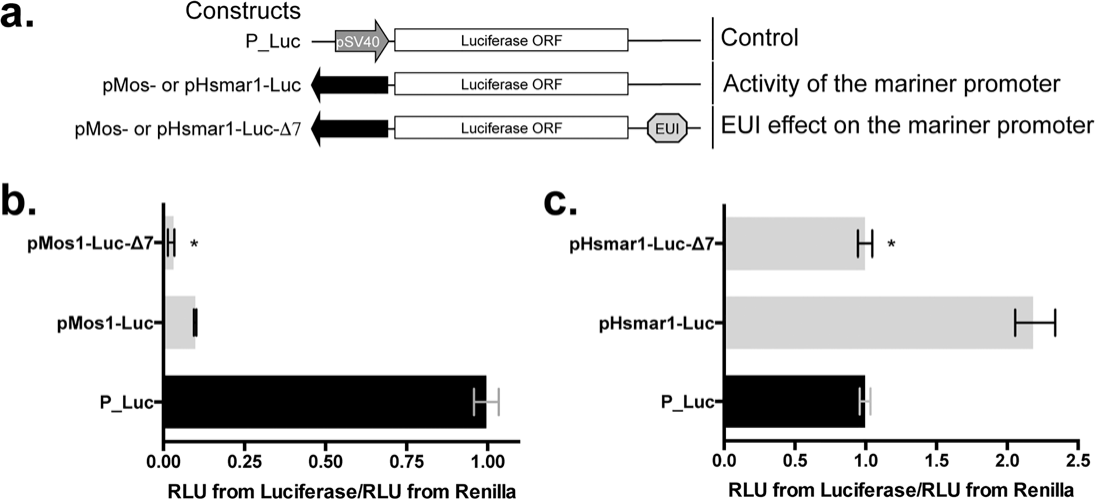
Activity of the *Mos1* and *Hsmar1* promoter in presence or absence of their Δ7 segment. (**a**) Schematic representation of expression cassettes containing a luciferase reporter gene that were used to evaluate the impact of the DNA element under investigation (EUI; here Δ7 segment) on their promoter pMos or pHsmar1 that are composed of the 5’ inverted terminal repeat plus the 5’ untranslated terminal region (black arrows). The DNA sequences of pMos and pHsmar1 are supplied in S3 Fig. The assay is based on the transient expression of two plasmids: (i) the pRL-Tk plasmid that expresses the *Renilla* luciferase under control of a Thymidine kinase promoter as a control for transfection efficiency, (ii) a derivative of the pGL3 plasmid that expresses the *Firefly* luciferase under control of an early SV40 promoter. Effect of Δ7-*MOS1* (**b**) and Δ7-*HSMAR1* (**c**) DNA segments on their own promoter in HeLa cells. Results are represented by median values from three experiments done in triplicate. Bars correspond to quartiles 1 and 3. * indicates significant differences (p<0.05) between the pMos1-Luc-Δ7, pHsmar1-Luc-Δ7, and pMos1-Luc or pHsmar1-Luc, respectively.

Taking into account the sequence of the active promoter in *Hsmar1* [15], a variant of transient luciferase expression assay was designed with luciferase expression plasmids containing the *Mos1* and the *Hsmar1* promoters (Figs 4A and S3). Our results with HeLa cells (Fig 4B and 4C) revealed that both promoters were active. pMos1 was found to be 10-fold less efficient than the early promoter for SV40 (pSV40) under these experimental conditions. pHsmar1 was found to be two-fold more active than the pSV40 contained in the P_Luc control. When their silencer were cloned downstream of the marker gene our results revealed levels of marker expression that were lower than those of the controls (3.3-fold for pMos1 and 2-fold for pHsmar1). This indicated that *mariner* silencers were able to negatively interfere with their own promoters. Because the closest transcriptional start site (TSS) upstream of the silencer element is that of the transposase ORF this mechanism is probably a way for MLEs to repress their transcriptional activity in their host cells and maintain active copies in a state of latency when host factors required for this repression are available.

In the next four sections we present the results of molecular and cellular biology investigations in which the *Mos1* silencer was used as the main model to elucidate the mechanism of its activity. The silencer of *Hsmar1*, and in a few cases those of *Himar1* and *Mcmar1*, were used as complements to confirm certain results. In the final section of the results pertaining to silencer activity at the scale of a eukaryotic genome, the *Hsmar1* and *Hsmar2* silencers were used, as they were the only models for which *in silico* genomic data are available.

### Mode of action of the *mariner* silencer element: Transcriptional control?

**Fig 5:**
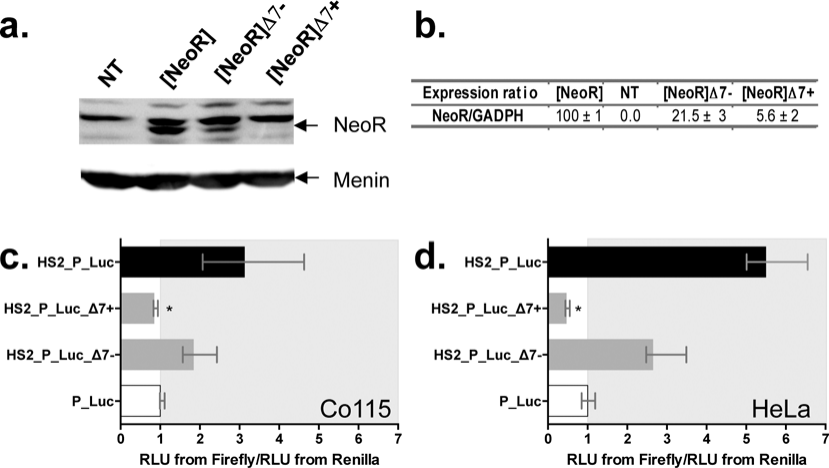
Steps for marker expression and factors involved in the silencing process. (**a**) Analysis of the NeoR protein expression by Western-blot 24 h post-transfection. The NeoR protein was revealed by hybridizing an antibody directed against the C-terminal end (Abcam 49973) in protein extracts from non-transfected HeLa cells (NT) or cells transfected with the [*NeoR*], [*NeoR*]Δ7- or [*NeoR*]Δ7+ constructs. A housekeeping protein, menin, was used as a positive constitutive expression control as described [16]. (**b**) mRNA expression of the *NeoR* gene by RT-qPCR. Total RNA extracts from the same cellular samples described in (**a**) were assayed. The endogenous *GAPDH* (Glyceraldehyde-3-phosphate dehydrogenase) gene was used as a positive constitutive expression control for normalization (*NeoR/GAPDH* ratios). The average expression obtained with the positive control [*NeoR*] was fixed at 100% and used as a reference to calculate the average expression of the 3 other extracts and the standard deviations. (**c**) and (**d**), expression of the *Firefly* and the *Renilla* luciferase marker genes using transient expression assays in cell lineages with specific profiles for TARBP2 and NRSF proteins. (**c**) Co115 (TARBP2-), (**d**) HeLa (TARBP2+) cells. Each histogram bar corresponds to the median value obtained from three experiments done in triplicate. Bars correspond to quartiles 1 and 3. The median ratios RLU from *Firefly*/RLU from *Renilla* were calculated as indicated in Fig 2. The area where the ratios “RLU from *Firefly*/RLU from *Renilla*” were above 1 (i.e. where no strong silencer effect is observed) is coloured in grey. * indicates a significant difference (p<0.05) with the P_Luc controls.

*NeoR* expression was monitored for 24 hours both at the protein and mRNA levels using cellular extracts from cells transiently transfected with our constructs. Western-blot analyses (Fig 5A) revealed that the amount of neomycin phosphotransferase 2 (NeoR protein) was ~5-fold lower in cells transfected with [*NeoR*]Δ7- than with [*NeoR*]. However, few or no NeoR protein was detected in cells transfected with [*NeoR*]Δ7+. This was also supported by RT-qPCR experiments (Fig 5B) which showed that there were respectively 5 and 20-fold fewer *NeoR* transcripts in cells transfected with [*NeoR*]Δ7- and [*NeoR*]Δ7+ than in those transfected with [*NeoR*]. Taken together these results confirmed that the *Mos1* silencer element interferes with the expression of a gene marker located immediately upstream, that it acts at the level of RNA, and that the strength of the effect depends on its orientation since the amount of *NeoR* transcripts in cells transfected with [*NeoR*]Δ7- was ~4-fold higher than in those transfected with [*NeoR*]Δ7+.

Because the silencer element had to be located within or downstream of the marker gene to be effective, we investigated whether it directly interfered with processes occurring after transcription initiation. Transcript quality and RNA interference were examined. Polyadenylation tails of transcripts from cells transfected with [*NeoR*], [*NeoR*]Δ7- and [*NeoR*]Δ7+ were investigated [17,18] according to their concentration in each sample, using *GAPDH* transcripts as endogenous controls. No difference was found, indicating that polyadenylation was unlikely to be affected. To test if the miRNA pathway was involved, Co115 human cells depleted in a key protein for miRNA processing and DICER function TARBP2 [19] were used in a transient expression assay. Similar silencing activity of [*NeoR*]Δ7+ was found in both Co115 (Fig 5C) and HeLa cells (Fig 5D), suggesting that there was no link between the silencing activity of Δ7 and the miRNA pathway.

### Mode of action of the *mariner* silencer element: A YY1 mediated control?

**Fig 6:**
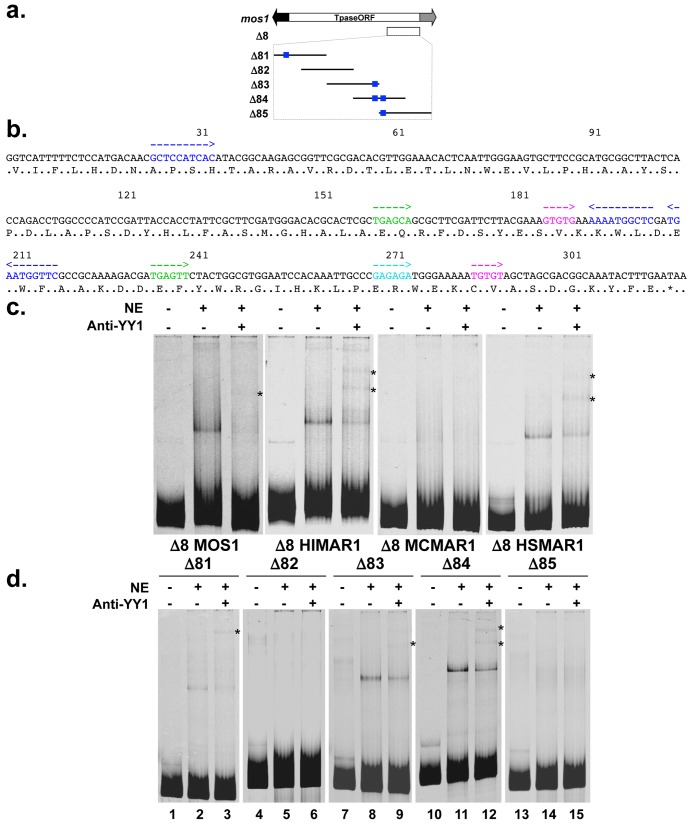
Characterization of the YY1 binding sites within the Δ8 segments. (**a**) Schematic representation of the *Mos1* transposon and its 10 deletion derivatives used to define a minimal silencer (Δ8 and Δ81 to Δ89). Δ8 spanned from positions 903 to 1212 of the *Mos1* sequence. Five derivatives of ~100 bp were tiled over ~50 bp (Δ81 to Δ85). Blank rectangle: *Mos1* transposase (Tpase ORF). Black arrow: 5’ ITR fused to the 5’ UTR. Grey arrow: 3’ UTR fused to the 3’ ITR. (**b**) Nucleic acid sequence of the Δ8-*MOS1* segment and putative TF binding sites frequently found within PRE in drosophila. Blue: *YY1*, red: *NRSF*, green: *Zeste*, pink: *GAGA* factor, turquoise: *GTGT* factor. Arrows indicate their orientation. The amino acid sequence of the C-terminal region of MOS1 is indicated using the one-letter code. (**c**) DNA binding activity of the YY1 factor to the Δ8-*MOS1*, Δ8-*HIMAR1*, Δ8-*MCMAR1*, and Δ8-*HSMAR1* segments. HeLa cells nuclear extracts were incubated with the ATTO-labelled Δ8 segments of the four transposases and the DNA-protein complexes were visualized by EMSA photography. Specificity was assayed using an anti-YY1 serum and super-shifted YY1/Δ8 complexes (one or two bands) are indicated by an asterisk. The content of each sample is shown above each lane: NE: nuclear extract, Anti-YY1: anti-YY1 serum. (**d**) Detection of YY1 binding sites within the Δ81 to Δ85 segments. The specificity of the shifted complex observed in lane 2, 8 and 11 was verified in lane 3, 9 and 12 in which the anti-YY1 serum allows obtaining super-shifted complexes (indicated by *). All these experiments were done in triplicate and representative pictures are shown.

In an attempt to locate a smaller fragment that would keep silencing activity in our expression assays the Δ7-*MOS1* segment was fragmented (Fig 6A). The Δ8-*MOS1* segment, corresponding to the last 317 bp of the *MOS1* ORF was found to have the same silencing activity as the Δ7-*MOS1* segment (S4 Fig). The size of the Δ8 segment is of interest since it was close to the upper limits for a usable protein electrophoretic mobility shift assays (EMSA) for investigating the binding of a TF.

Several viruses and transposable elements [20–30] were previously found to contain segments capable of silencing their own transcriptional activity to establish their latency in their eukaryotic hosts. These silencers are bound by the transcription factor Yin Yang 1 (YY1 in vertebrates, Pho in drosophila). In eukaryotes, YY1 and other TFs can bind a chromosomal polycomb response element (PRE) to mobilize the PRC1 and PRC2 and finally induce transcriptional silencing of that chromosomal region.

The presence of YY1 binding sites and TF binding sites involved in PRC2 in drosophila was examined in the Δ7 and Δ8 silencer segments of *Mos1* (Fig 6B), *Himar1*, *Mcmar1*, *Hsmar1*, and *Hsmar2* (S5 Fig). A set of binding sites for YY1 or Pho, the GAGA factor (GAF), GTGT factor (GTF), and Zeste [31,32] located among the Δ7 segments was found in all natural MLEs, each of which is typically repeated. Together the presence of these sites suggests that PRCs might be able to bind to these silencer elements, at least in dipteran species.

EMSAs were carried out to verify the presence of functional YY1 binding sites in Δ8 *mariner* segments. Our results showed that a shifted complex was present with the Δ8-*MOS1*, Δ8-*HIMAR1* and Δ8-*HSMAR1* probes (Fig 6C). These complexes were super-shifted by anti-YY1 antibodies confirming that they correspond to YY1/Δ8 complexes. The absence of a complex with the Δ8-*MCMAR1* probe suggested that the binding site located in Δ8 was not bound under our experimental conditions. Hence, the silencing element in *Mcmar1* extended beyond Δ8 and might be located at the 5’ extremity of Δ7, which contains a YY1 binding site (S5 Fig).

Since only one shifted band was observed with the Δ8-*MOS1* segment while three binding sites were predicted in its sequence, further EMSA investigations were performed with shorter probes, Δ81 to Δ85 (Figs 6A and 6D and S4). These results were consistent with the sequence binding site prediction analysis, showing that there was one YY1 binding site within Δ81 and Δ83, two in Δ84, and none in Δ82 and Δ85. This last result suggested that the motif located in 3’ was unable to be bound by YY1 under our experimental conditions. However, this was likely an artefact due to its location at the 5’ end of the Δ85 probe. Indeed, when both YY1 sites are located in the middle of the Δ84 probe, two shifted bands were observed (Fig 6D, lane 12), suggesting that both sites could be bound. Taken together, these data supported the conclusion that the silencer activity of the Δ8 segments was possibly mediated by one or several YY1 silencing pathways.

### Other TFs binding to the *mariner* silencer element: The case of NRSF

Since the definition of PREs in vertebrate genomes is an issue that has yet to be fully elucidated [31,32], we searched for motifs conserved for both sequence and location using the MEME software suite with the DNA sequences of 34 *mariner* Δ7 segments (S6 Fig). A single conserved 30 bp motif was found (p-values ranging from 3.36e^-21^ to 6.11e^-14^) that spanned the region coding one of the two signature motifs o*f marin*er transposases, the PHxxYSPDLAPxD peptide [33], and located in the region as a putative non-cardinal binding site for NRSF [34,35] and charlatan [36]. In mammalian genomes, approximately 80% of the 2 000 characterized NRSEs (called RE1) correspond to a 21 bp motif consisting of two conserved motifs of 9 and 10 bp separated by a 2 bp linker. Approximately 12% consist of multiple rearrangements of this motif [34–36]. The remaining 8% are sites with no conserved motifs. The putative NRSE in the *mariner* silencer element described above belongs to the second category.

**Fig 7:**
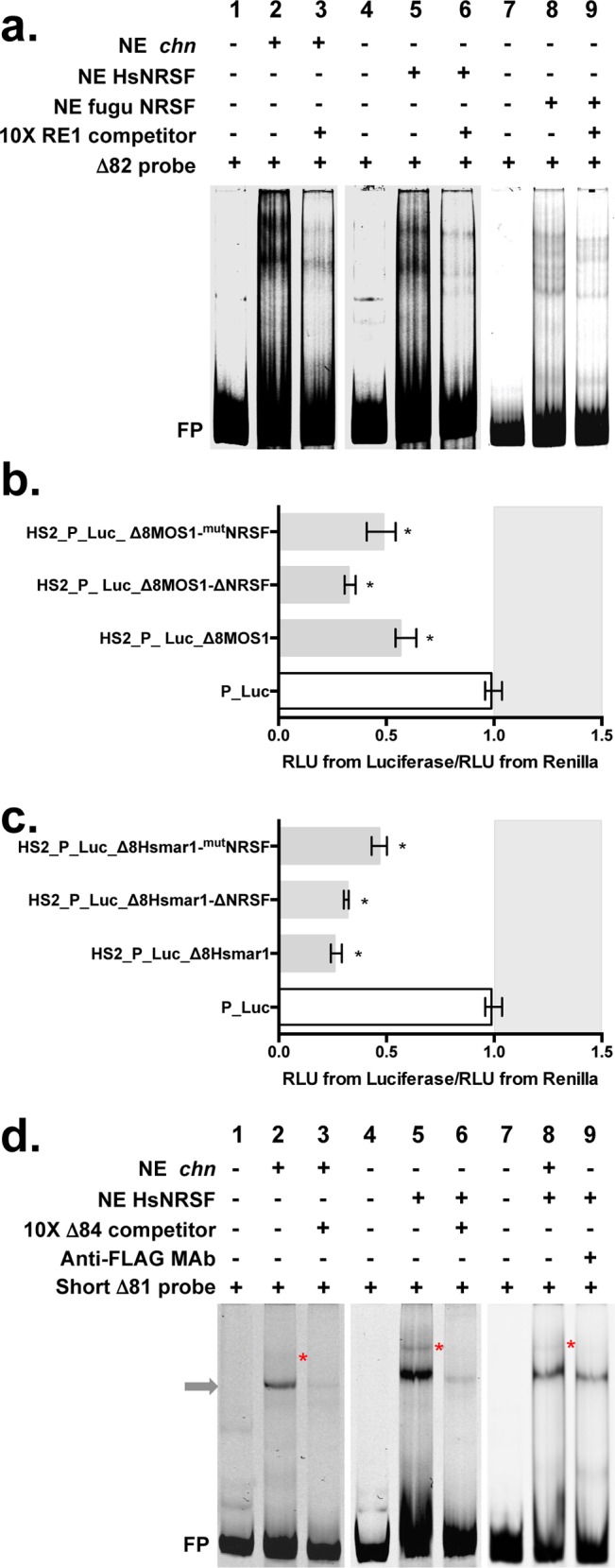
Binding of NRSF proteins within the Δ8 *mariner* DNA segments. **(a)** DNA binding of chn, HsNRSF, fuguNRSF to the Δ82 segment of *MOS1* (10 pmole of ATTO-labelled probe/lane). Lanes 1, 4 and 7 correspond to probe controls. Lanes 2, 5 and 8 show shifted complexes with each of the three NE. The specificity of the shifted complexes observed in lanes 2, 5 and 8 was verified by adding 10X of unlabelled RE1 DNA segments (lanes 3, 6 and 9). These experiments were done in triplicate and representative pictures are shown. (**b**) and (**c**) Expression of the *Firefly* and the *Renilla* luciferase marker genes using transient expression assays in HeLa cells. The assays were performed with three variants of Δ8-*MOS1* in **c** and Δ8-*HSMAR1* in **d**. The sequence of these variants is supplied in S11 Fig. Each histogram bar corresponds to the median value obtained from three experiments done in triplicate. Bars correspond to quartiles 1 and 3. The median ratios RLU from *Firefly*/RLU from *Renilla* were calculated as indicated in Fig 2. The area where the ratios “RLU from *Firefly*/RLU from *Renilla*” were above 1 (i.e. where no silencer effect is observed) is coloured in grey. * indicates a significant difference (p<0.05) with the P_Luc controls. (**d**) DNA binding of chn and HsNRSF to a shortest version of the Δ81 segment of *MOS1* (10 pmole of ATTO-labelled probe/lane; Sequence supplied in S4 Fig). Lanes 1, 4 and 7 correspond to probe controls. Lanes 2, 5 and 8 show shifted complexes with each of the two NEs. The grey arrow locates a complex resulting from the binding of YY1, as shown in Fig 6D. The specificity of this YY1 complex in lanes 2 and 5 was verified by adding 10X of unlabelled Δ84 DNA segment that contains two YY1 binding sites. The red stars in lanes 2, 5 and 8 locate a complex that is absent when HeLa NE (Fig 6D) are used. The involvement of HsNRSF in this second complex is shown in lane 9, using a specific antibody that leads to its destabilization. It should be noted that this second complex is also sensitive to competition with the unlabelled Δ84 DNA segment (lanes 3 and 6).

EMSAs were carried out to assay these predicted NRSEs. HeLa nuclear extracts containing charlatan, human or fugu NRSF tagged with FLAG or Myc were prepared as described in previous studies [36,37]. The activity of the nuclear extracts was validated using an NRSE probe (RE1) in EMSA (S7 Fig) carried out with appropriate competitor and/or antibodies [36–38]. The binding of NRSF to Δ8-*MOS1* was then further investigated with EMSA using shorter versions of Δ8, Δ81 to Δ85 segments as probes and HeLa nuclear extracts containing charlatan, human or fugu NRSF tagged with FLAG or Myc. No shifted bands were obtained with Δ83, Δ84 and Δ85 probes. By contrast, shifted complexes sensitive to the competition by a specific competitor (unlabelled RE1 fragment) were obtained with the Δ82 probe for the three NRSF proteins (Fig 7A). Since this probe contained the motif encoding the PHxxYSPDLAPxD peptide, we concluded that it was an NRSE.

To verify whether these NRSE intervened in the silencer activity under our experimental conditions, transient luciferase expression assays were performed using constructs with the Δ8, Δ8-Δ*NRSF*, or Δ8-^*mut*^*NRSF* of *Mos1* or *Hsmar1* (S8A and S8B Fig) cloned in positive orientation downstream of the marker cassette of HS2_P_Luc plasmids (Fig 7B and 7C). Δ8-*MOS1*-Δ*NRSF* and Δ8-*HSMAR1*-Δ*NRSF* were specified by the deletion of the NRSE motif and Δ8-*MOS1*-^*mut*^*NRSF* and Δ8-*HSMAR1*-^*mut*^*NRSF* by the mutagenesis of the NRSE by randomly shuffling its sequence. Results revealed that the DNA motif encoding the PHxxYSPDLAPxD peptide, i.e. the NRSE, was not essential for the silencer activity of the Δ8 silencers of *Mos1* and *Hsmar1*.

**Fig 8:**
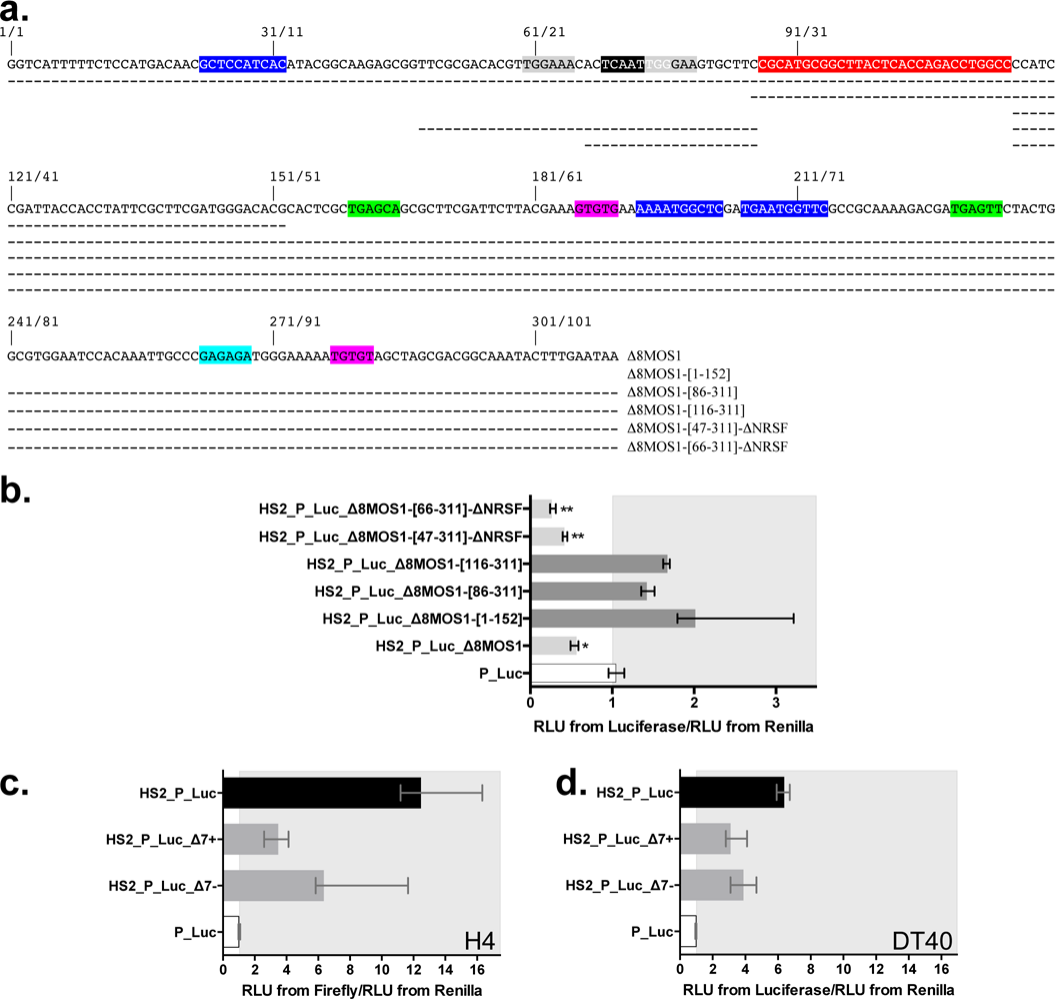
Location of regions essential to silencing activity within the Δ8 segments of *MOS1*. (**a**) Variant fragments within the sequences of the Δ8-*MOS1*. Dashes indicate positions that are present in each fragment. (**b**) Expression of the *Firefly* and the *Renilla* luciferase marker genes using transient expression assays in HeLa cells. The assays were performed with Δ8-*MOS1* and five variants cloned in + orientation. (**c**) and (**d**) Impact of the Δ7 segments of *MOS1* on the expression of the *Firefly* and the *Renilla* luciferase marker genes using transient expression assays in cell lineages H4 (TARBP2+/NRSF-/YY1+/NFAT-5-) (**c**) and DT40 (TARBP2+/NRSF+/YY1+/NFAT-5-) (**d**). In (**b**), (**c**) and (**d**), each histogram bar corresponds to the median value obtained from three experiments done in triplicate. Bars correspond to quartiles 1 and 3. The median ratio RLU from *Firefly*/RLU from *Renilla* were calculated as indicated in Fig 2. The area where the ratios “RLU from *Firefly*/RLU from *Renilla*” were above 1 (i.e. where no strong silencer effect is observed) is coloured in grey. In (**b**) * and ** indicate a significant difference (p<0.05) with the P_Luc controls. ** also indicates a significant difference (p<0.05)with HS2_P_Luc_Δ8-*MOS1*.

Two shifted complexes were also obtained with the three NRSF proteins and the short Δ81 probe in which there was one YY1 binding site and no overlap with the *NRSE* encoding the PHxxYSPDLAPxD peptide (Fig 7D lanes 2, 5 and 8; S9 Fig, lanes 2 and 5). These two complexes were sensitive to competition by a specific competitor of the YY1 binding (Fig 7D, lanes 3 and 6; S9 Fig, lane 3), indicating that they involved YY1. Interestingly, we observed that the bigger complex (indicated by a red star in Figs 7D and S9) disappeared when antibodies directed against the tag of the human or fugu NRSF were added (Fig 7D, lane 9; S9 Fig, lane 6). Together these results indicated that there was a second NRSF binding site in the Δ8 silencer that required the cooperative binding of YY1 to be efficient. In spite of our efforts we failed to locate an NRSE or a charlatan binding element in this region. Therefore, it remains possible that NRSF only interacts with YY1 when it is bound to Δ8. In order to verify whether this second site of NRSF binding was required for the silencer activity two Δ8 variants for *Mos1* (Fig 8A) and *Hsmar1* (S8B Fig) were generated by PCR, cloned downstream of the marker gene into HS2_P_Luc plasmid constructs in positive orientation, and tested in transient luciferase expression assays in HeLa cells. Results obtained with constructs HS2_P_Luc_Δ8-*MOS1*-[47–311]-Δ*NRSF* (Fig 8B) and HS2_P_Luc_ Δ8-*HSMAR1*-[61–311]-Δ*NRSF* (S8C Fig) revealed that the silencer activity was conserved in spite of the fact that both regions bound by NRSF proteins in *Mos1* were deleted in Δ8 segments.

Finally, these data supported that there were either one or two sites where NRSF was able to interfere with the Δ8 segment. However, the binding of NRSF to the *mariner* silencers of *Mos1* and *Hsmar1* was not essential to the silencer activity under our experimental conditions. Therefore, we continued our efforts to find out the regions essential for the silencer activity of the *mariner* Δ8 segment.

### DNA regions essential for the silencer activity

In addition to the variant Δ8-*MOS1*-[47–311]-Δ*NRSF*, four other variants were made (Fig 8A). The first, Δ8-*MOS1*-[1–152], contained the 5’ half of Δ8-*MOS1* (i.e. one YY1 binding site plus the two NRSF binding sites). The second, Δ8-*MOS1*-[86–311], contained the 3’ half plus the NRSF binding site overlapping the DNA motif encoding the PHxxYSPDLAPxD peptide (i.e. two YY1 binding sites plus one NRSF binding site). The third, Δ8-*MOS1*-[116–311] was similar to the second with the exception that its NRSF binding site was removed. The fourth, Δ8-*MOS1*-[66–311]-Δ*NRSF*, was similar to Δ8-*MOS1*-[47–311]-Δ*NRSF* but its 19 residues on the 5’ end were deleted.

Transient luciferase expression assays in HeLa cells revealed that all of these Δ8 variants had kept their silencer activity but with variable efficiency (Fig 8B). For Δ8-*MOS1*-[1–152], Δ8-*MOS1*-[86–311] and Δ8-*MOS1*-[116–311], the luciferase expression is higher than the P_Luc control but, significantly, it was 2.5 to 3.5-fold lower than that of HS2_P_Luc (e.g. in Figs 2A and 5B). This indicated that the YY1 binding site motif within the 5’ half of Δ8 and other TF binding sites within the positions 116 to 311 were enough to trigger weak silencer activity in HeLa cells. Interestingly, it also indicated that Δ8-*MOS1*-[47–311]-Δ*NRSF* and Δ8-*MOS1*-[66–311]-Δ*NRSF* had better silencer activity than Δ8 in HeLa cells. Taken together, these results suggested that several combinations of TFs could bind to Δ8 and could cooperatively act with YY1 to trigger the silencer activity.

To verify whether this property could be recovered in another *mariner* silencer, Δ8 variants were also made from Δ8-*HSMAR1* (S8B Fig). Their analysis under similar experimental conditions first revealed that the 3’ half (positions 130 to 310) was enough to trigger weak silencer activity in HeLa cells, but was more efficient when the DNA motif bound by NRSF and overlapping the DNA motif encoding the PHxxYSPDLAPxD peptide was present (positions 86 to 310). This indicated that this NRSF binding site favoured the silencer activity in Δ8-*HSMAR1*. In addition, the two variants Δ8-*HSMAR1*-[61–310]-Δ*NRSF* and Δ8-*HSMAR1*-[81–310]-Δ*NRSF* that have sequence properties similar to those of Δ8-*MOS1*-[47–311]-Δ*NRSF* and Δ8-*MOS1*-[66–311]-Δ*NRSF* have kept a strong silencer activity, but this was significantly less strong than that of Δ8.

A search for TF binding sites motifs within the regions 47 to 96 in Δ8-*MOS1* and 61 to 104 in Δ8-*HSMAR1* was achieved using the MatInspector facilities of the GENOMATIX software suite (Munich, Germany). Our results revealed that there were two NFAT-5 and one Alx1 binding sites in the 50 bp *MOS1* segment (Fig 8A), and one NFAT-5 and one Alx1 binding sites in the 44 bp *HSMAR1* segment (S8B Fig). Under the hypothesis that the same TFs acted on this region, results obtained with Δ8-*MOS1*-[47–311]-Δ*NRSF* and Δ8-*MOS1*-[66–311]-Δ*NRSF* on the one hand, and Δ8-*HSMAR1*-[61–310]-Δ*NRSF* and Δ8-*HSMAR1*-[81–310]-Δ*NRSF* on the other hand, suggested that both these TFs might cooperatively intervene in the silencer activity.

In order to further investigate this feature the HeLa, Co115, H4 and DT40 cells used in our work were phenotyped in order to determine their expression for *NRSF*, *YY1*, *NFAT-5*, *Alx1* and *TARBP2*. We found that HeLa cells were NRSF +, YY1 +, NFAT-5 +, Alx1 + and TARBP2 +. Others cells presented differences since Co115 cells were TARBP2 -, DT40 were NFAT-5 -, and H4 were NRSF—and NFAT-5 -. Taking into account these phenotypes, the effect of the Δ7-*MOS1* segments in transient luciferase expression assay was analyzed (Fig 5C and 5D and Fig 8C and 8D). Under these experimental conditions the absence of NFAT-5 significantly weakened the silencer effect of Δ7-*MOS1* in H4 and DT40, but did not suppress it entirely.

In conclusion, our results suggested that TFs NFAT-5, Alx1 and NRSF, might intervene alone or cooperatively with YY1 to bind to the silencer of *Mos1* and *Hsmar1* and elicit the silencer activity. In addition, the weak silencer activity of the region located downstream of the DNA motif encoding the PHxxYSPDLAPxD peptide of Δ8-*MOS1* and Δ8-*HSMAR1* might be related to the cooperative binding of YY1 and GAF and/or GTF TFs.

### Involvement of PRC2 with *mariner* silencers

**Fig 9:**
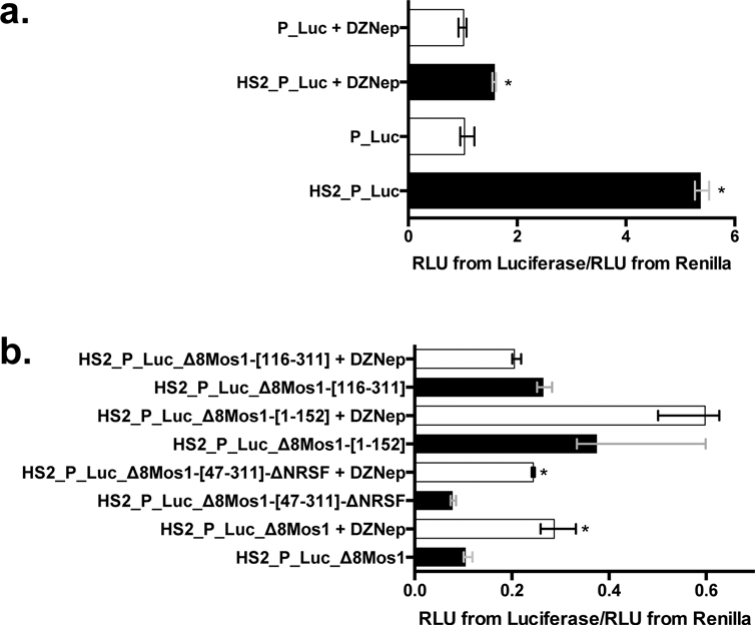
Impact of PRC2 inhibition with DZNep on the silencer of *Mos1*. (**a**) Expression of the *Firefly* luciferase marker gene from P_Luc and HS2_P_Luc in absence or presence of 5 μM DZNep using transient expression assays in HeLa cells. (**b**) Expression of the *Firefly* luciferase gene from 4 different HS2_P_Luc plasmid constructs containing Δ8-*MOS1* variants cloned downstream of the marker gene. In (**a**) and (**b**), each histogram bar corresponds to the median value obtained from three experiments done in triplicate. Bars correspond to quartiles 1 and 3. * indicates a significant difference (p<0.05) between cells treated or not with 5 μM DZNep. In (**a**), the ratio “RLU from *Firefly*/RLU from *Renilla*” for the P_Luc transfection in the absence of DZNep is used as a reference and fixed at 1 arbitrary unit. All other ratios were calculated taking this reference into account. In (**b**), the ratios “RLU from *Firefly*/RLU from *Renilla*” for the HS2_P_Luc transfection done in absence or presence of 5 μM DZNep are used as references and fixed at 1 arbitrary unit. They were respectively used to calculate the ratios of each construct assayed in absence or in presence of 5 μM DZNep.

The sequence features of the Δ8 *mariner* silencers described above might match those of the Polycomb Responsive Elements/Trithorax Responsive Elements (PRE/TRE) [31,39–42] that respectively silence or activate gene transcription by modifying chromatin histone marks. In order to further investigate whether the silencing depended on the polycomb pathway we used a specific inhibitor of PRC2, the 3-deazaneplanocin A (DZNep), in expression assays using plasmid constructs containing different variants of the *mariner* silencers of *Mos1* (Fig 9) and *Hsmar1* (S10 Fig). DZNep is an analogue of 3-deazadenosine that inhibits the activity of S-adenosylhomocysteine hydrolase, leading to the indirect inhibition of various S-adenosylmethionine-dependent methylation reactions, such as those catalysed by EZH2 in animal cells, including HeLa cells [43–45]. DZNep efficiently inhibits EZH2 after 8 h of treatment and can induce strong apoptotic cell death reaction in cancer cells beyond 48 h [43–47]. Cells were treated overnight with 5 μM DZNep prior DNA transfection and treatment was maintained until *Firefly* and *Renilla* luciferase activity measurements.

Before experimenting with DZNep on *mariner* silencers, the impact of this chemical was verified on the *Firefly* luciferase expression of the P_Luc and HS2_P_Luc constructs (Fig 9A). Results revealed that DZNep had no effect on P_Luc. However, this chemical decreased the capacity of the HS2 enhancer to boost the *Firefly* luciferase expression (~3.5-folds) even if the difference between P_Luc and HS2_P_Luc constructs remained significant. Because our silencer DNA segments were cloned into an HS2_P_Luc plasmid backbone, expression results obtained with HS2_P_Luc in the presence or absence of 5 μM DZNep were used as references to calculate the expression rate obtained with the *mariner* silencer constructs in the presence or absence of 5 μM DZNep (Fig 9B and S10 Fig).

Results showed that DZNep significantly increased (p<0.05) the *Firefly* luciferase expression from HS2_P_Luc_Δ8-*MOS1*, HS2_P_Luc_Δ8-*MOS1*-[47–311]-Δ*NRSF*, HS2_P_Luc_Δ8-*HSMAR1*, HS2_P_Luc_Δ8-*HSMAR1*-Δ*NRSF* and HS2_P_Luc_Δ8-*HSMAR1*-[61–310]-Δ*NRSF*. This supported the hypothesis that the *Mos1* and *Hsmar1* might contain functioning silencers depending on the PRC2 pathway, since the silencer activity of these constructs is decreased. Interestingly, five constructs responding to a DZNep treatment shared the presence of YY1, NFAT-5, Alx1, GAF and GTF binding sites in their DNA sequences.

By contrast, the *Firefly* luciferase expression from constructs containing the 5’ half of the *Mos1* silencer (HS2_P_Luc_Δ8-*MOS1*-[1–152]) or the 3’ half of the *Mos1* or *Hsmar1* silencers (HS2_P_Luc_Δ8-*MOS1*-[116–311] and HS2_P_Luc_Δ8-*HSMAR1*-[115–310]) were not affected by the DZNep treatment. This suggested that the weak silencer effect resulting from the presence of these DNA segments might result from another silencing mechanism and is detected only when PRC2 is disrupted. Such a duality between silencing pathways was previously described for *ITm* TEs contained in the genome of murine ES cells. Indeed, these TEs can switch from heterochromatinization mediated by the HP1 (Heterochromatic Protein 1) dependent pathway to a PRC2-dependent silencing when the Histone-lysine N-methyltransferase Su(var)39/HP1 is disrupted [29]. Here, the duality between silencing pathways might also help explain why weak residual silencer effects were observed in some cases, such as in the H4 and DT40 cells (Fig 5C and 5D).

### Epigenetic status of MLEs in the human genome

Since there is no available animal model with active MLEs for which high throughput chromatin data are available in public databases, we have investigated the chromatin status of two human MLEs, *Hsmar1* and *Hsmar2*, that appeared in the human genome approximately 50 and at least 80 million years ago respectively [48,49]. Currently these elements have lost their ability to transpose due to the accumulation of nucleotide mutations in the ORF coding for their transposase. The advantage of the human model is that it has the richest set of ChIP-Seq data for TFs and histone modifications. Because the recruitment of TFs bound to DNA at the moment of the establishment of histone modifications is not subsequently required for their maintenance and transmission over cell divisions [50–53], we have focussed our investigations on histone modifications. This was carried out in order to highlight potential associations between the presence or the absence of a complete *mariner* silencer within each human MLE, their genomic location, and two important silencing pathways: polycomb/trithorax and Su(var)39/HP1. These two pathways lead to specific signatures of histone modifications: (i) H3K27me3 when the genomic loci is inactivated by PRC, (ii) H3K27me3/H3K4me3 when PRC and Trithorax complexes interfere together at level of inactive poised regions, (iii) H3K27ac/H3K4me3 when the genomic loci is activated by Trithorax complexes, and (iv) H3K9me3 and H4K20me1 when it is silenced and heterochromatinized by the Su(var)39/HP1 pathway [39,41,54,55]. Since it was previously shown that human TEs carry more histone modifications when they are located within or near genes [56], we have distinguished two categories of MLEs: those located in genes coding for proteins and those in inter genic regions. As a first step in our analysis, we inventoried the sequence features of *Hsmar1* and *Hsmar2* in the human genome using the hg19 RepeatMasker annotation (S11 Fig, S1 Table). Among the 592 and 1240 loci containing respectively an *Hsmar1* or an *Hsmar2* segment, 361 and 595 contained a nearly full-length copy and 315 and 644 contained *Hsmar1* and *Hsmar2* Δ8 silencers. Their chromatin status (Polycomb (P), Trithorax (T), Su(var)39/HP1 (H) or a mix of these statuses) was then inventoried in 14 human cell lines using CHIP-seq peaks (S2 Table; S12 Fig). In a second step, an analysis of the chromatin status was carried out at the scale of complete populations of *Hsmar1* and *Hsmar2* using a silencer definition in which the sequence of Δ8 segments was complete, absent, or damaged (Sil+, Sil- and U) and their genic or inter genic location in the human genome (S1 Table). Results indicated that the chromatin status was only statistically defined for 25 to 71% of loci, depending on the cell type and the features of the *mariner* element (S2 Table). Statistical analyses were carried out to test putative associations between the chromatin status, the presence of a silencer, and their genome location (S1 Table). A Wilcoxon test verified the associations between the percentage of Sil+ and Sil- and the polycomb status in cell lines. A Student t-test was used to search for associations between the quantity of polycomb status in Sil+ and Sil- loci. Only one robust association was found with both tests for *Hsmar2*. It revealed that *Hsmar2* Sil+ has significantly more often a polycomb status than *Hsmar2* Sil- in genic regions (p value = 0.00428 with the Wilcoxon signed-rank test and 0.02084 with the t-test, see methods). Features of *Hsmar1* and *Hsmar2* elements were therefore further investigated in order to i) verify whether genomic *Hsmar1* silencer were still active and ii) verify whether the propensity of at least a part of *Hsmar2* Sil+ to have a polycomb status was due to their activity.

**Fig 10:**
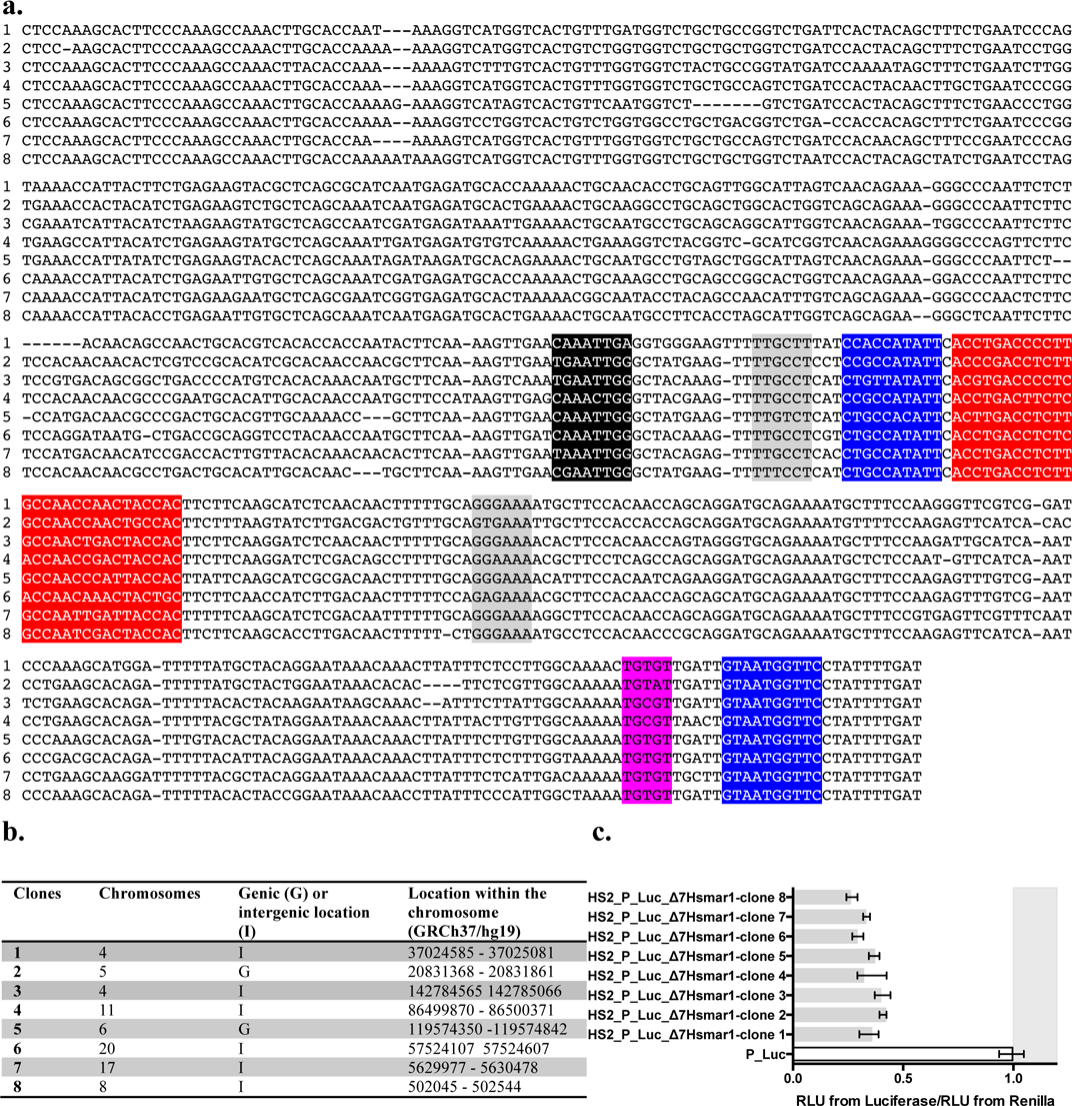
**Sequences (a), chromosomal locations (b) and silencer activity (c) of eight Δ7 *Hsmar1* segments amplified from human DNA and sub-cloned.** In (**a**), conserved DNA binding motifs described in Fig 6B are highlighted in red for NRSF, blue for YY1 or Pho, pink for GTGT factor, grey for NFAT-5, and black for Alx1, respectively. Clones were amplified by PCR using human gDNA and primers (5’-CTCCAAAGCACTTCCCAAAGC-3’ and 5’-ATCAAAATAGGAACCATTAC-3’) designed from the reference sequence HSU52077 (lane c in (**a**)). In (**c**), impact of 8 *Hsmar1* Δ7 segments on the expression of the *Firefly* and the *Renilla* luciferase marker genes using transient expression assays in HeLa cells. The median ratios RLU from *Firefly*/RLU from *Renilla* were calculated as indicated in Fig 2. Bars correspond to quartiles 1 and 3. The area where the ratios “RLU from *Firefly*/RLU from *Renilla*” were above 1 (i.e. where no strong silencer effect is observed) is coloured in grey.

We verified that at least a part of the *Hsmar1* elements still contained an active silencer because remnants of human MLEs had accumulated significant amounts of mutations due to their age (S11 Fig). Eight *Hsmar1* Δ7 segments were amplified by PCR from human gDNA, sub-cloned, sequenced, and located in hg19 (Fig 10A and 10B). These *Hsmar1* Δ7 segments were then assayed with our stable expression and transient expression assays to verify their silencer activity. All of them were found to be strong silencers (*luciferase/renilla* ratio > 0.5; p<0.05; Fig 10C). Their putative co-localizations with CHIP-seq peaks on their Δ8 moiety were investigated and our results showed that the chromatin status was statistically defined for 59% of cases (S3 Table). They suggested that 50–56% of the 8 loci had a Su(var)39/HP1 status, whatever the cell type and the loci, the other 44–50% having polycomb status. In agreement with the literature [42], this suggested that the impact of these 8 strong silencers on the local chromatin status in somatic cells mainly depended on their genomic environment and the origin of the cells. If *Hsmar1* silencers play a role in their host genome, our hypothesis is that they would intervene in chromatin organization during development or cell differentiation but not in adult somatic cells.

Because lacking data about the chromatin status (see loci with an undetermined chromatin status (ucs) in S1 Table) of human *mariner* silencers prevented the calculation of heat maps, only Sil+, Sil- and U located in intragenic regions and being annotated in at least seven cell lines were selected (187 loci, 95 Sil+, 67 Sil- and 25 U) to generate a heat map of the chromatin status *Hsmar2* silencers (S13 Fig). Both cladograms on the top and the left of the heat map indicated that there was a suitable segregation of loci which were preferentially associated with a polycomb (green box) or a Su(var)39/HP1 status (yellow box), excepted for H1-hESC. This observation about hESC was in agreement with previous works indicating that hESC had a global chromatin status that is less marked than in somatic adult cells [57]. This heat map also allowed locating loci with a bivalent status (P/T loci in the blue box and P/H loci in the orange boxes). In agreement with our previous results, we observed that the density of *Hsmar2* Sil+ loci associated with a polycomb status (91.5% of intragenic Sil+) was significantly above that of Sil- (73% of intragenic Sil- and U). Reciprocally, the density of Sil- associated to a Su(var)39/HP1 status (27% of intragenic Sil- and U) was significantly above that of Sil+ (8.5% of intragenic Sil+). Results with intragenic Sil- and U therefore suggested that only 20% of the *Hsmar2* Sil+ would have a chromatin status depending on the activity of their silencer.

**Fig 11:**
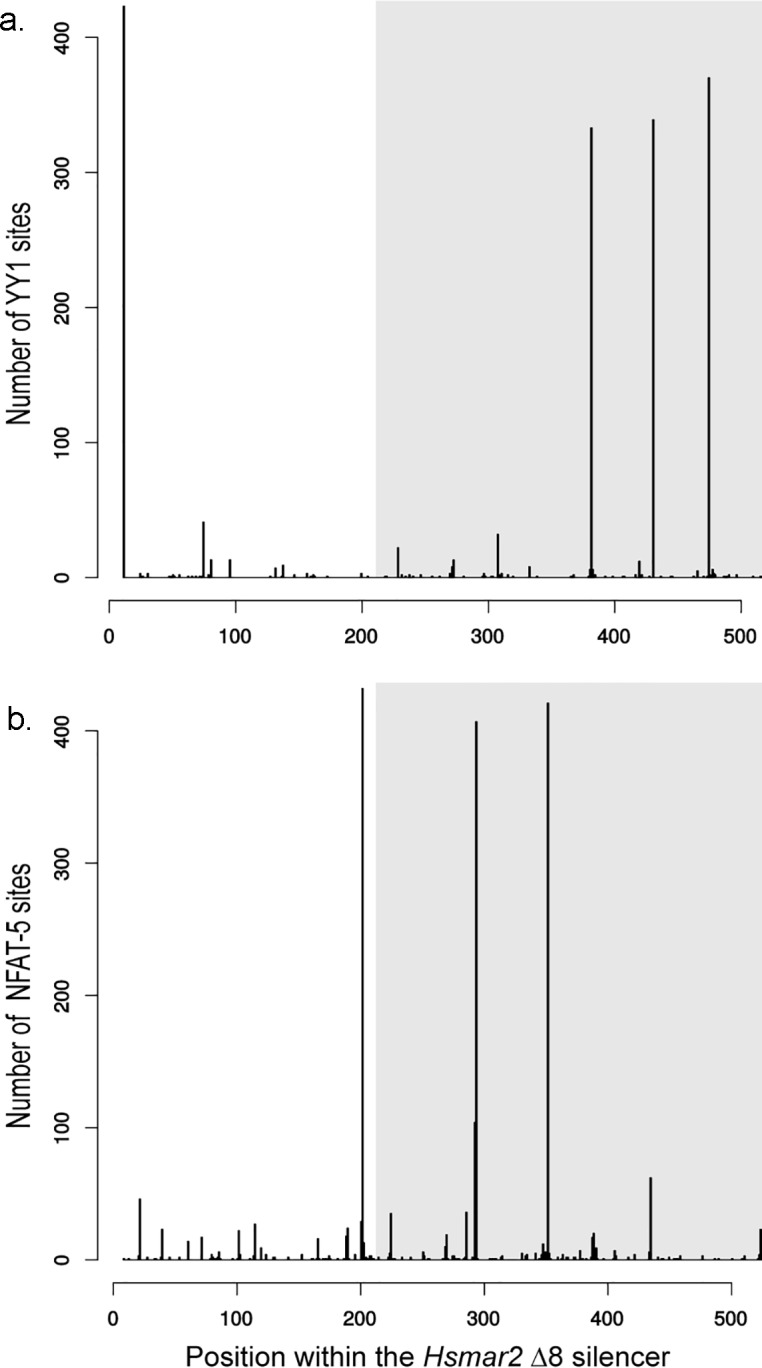
**Counting of YY1 (a) and NFAT-5 (b) binding sites along the sequence of genomic Δ7 *Hsmar2* segments.** Areas filled in grey located the Δ8 segment within the Δ7 *Hsmar2* segment.

To verify whether the propensity of intragenic *Hsmar2* Sil+ to be polycomb was due to their activity, we verified whether certain YY1 and NFAT-5 binding sites were significantly associated to the polycomb phenotype, taking into account that at least 1 YY1 and 1 NFAT-5 binding sites are required in an active *mariner* PRE. Because no result was statistically significant with the YY1 sites of Δ8 regions, sequences were extended in 5’ in order to match with a Δ7 segment. The YY1 and NFAT-5 binding sites were located in all *Hsmar2* loci with a segment Δ7. In agreement with the *Hsmar2* consensus sequence (S5 Fig), we found four YY1 binding sites at positions 11, 382, 431 and 475 (Fig 11A) of Δ7 segment and three NFAT-5 at positions 202, 293 and 294 (Fig 11B), all well conserved in numerous elements. For each binding site, a Wilcoxon test was used to verify the association between its presence and the propensity to have polycomb status in various cell lines. These tests revealed that the YY1 binding site at position 11 and the two NFAT-5 binding sites at positions 202 and 352 were significantly associated to loci with a polycomb status (p value = 0.014, 0.019 and 0.023 with the Wilcoxon signed-rank test, respectively). In agreement, the association of the YY1 site and one of the two NFAT5 sites in silencers was found to be significantly associated to loci with a polycomb status (p value = 0.017 with the Wilcoxon signed-rank).

Together, these results indicated that numerous intragenic *Hsmar2* elements displaying the Δ7 region would contain a silencer still active in somatic cells. These results also confirmed that the size of a minimal *mariner* silencer was variable and depended on the MLE “species”. It corresponded to the Δ7 region in *Hsmar2* and *Mcmar1* (S5 Fig) and only to the Δ8 region in *Himar1*, *Hsmar1* and *Mos1*.

## Discussion

Our work demonstrates that among all the MLEs we analysed all contain a silencer element within the last 300 to 500 bp of the transposase ORF. Under our experimental conditions the assayed silencers were found to have an optimal gene silencing effect when they were located from just downstream of the marker gene TSS to a few kpbs downstream of the gene transcription arrest site. We found that *mariner* silencers were able to silence strong (pCMV and pIE1), moderate (pSV40 and pHsmar1) and weak (pMos1) promoters, and were restricted by the host origin suggesting that they likely function with TFs that are conserved among animal species. Finally, our results support that *mariner* silencers largely function by promoting the PRC2 pathway, but they might also be able to trigger an alternate silencing pathway when PRC2 cannot be activated.

### TF candidates eliciting the activity of *mariner* silencers

In agreement with our hypothesis that *mariner* silencers function with TFs conserved among animal species we found that their activity may depend on the binding of at least five TF candidates and YY1. The binding of NFAT-5 alongside with YY1 (NFAT in *D*. *melanogaster*) to the *Mos1* and *Hsmar1* silencers is likely the key for the activity of *mariner* silencers. Alx1 (Php13-Hazy in *D*. *melanogaster*) and NRSF are also be able to promote the silencer activity but with lower efficiency. In addition, expression data obtained with HS2_P_Luc_ Δ8-*MOS1*-[116–311] (Fig 8A and 8B) indicate that Δ8-*MOS1*-[116–311] keeps a weak silencer activity in spite of the absence of the NFAT-5, Alx1, and NRSF sites, and the YY1 site located near the 5’ end of the Δ8-*MOS1* segment. This supports the conclusion that other TFs, such as GAF and-or GTF, might intervene in the silencer activity by binding to the 3’ half of the Δ8 segments (Figs 6B and S6). Since none of the Δ8-*MOS1* and Δ8-*HSMAR1* variants lost their silencer activity completely, it suggests that NFAT-5, Alx1, NRSF, GAF and GTF might function alone or more likely cooperatively with YY1 to trigger the silencing activity, depending on the cellular context. It should be noted that the variations in silencing efficiency of the Δ8-*MOS1* and Δ8-*HSMAR1* variants must be carefully considered. Indeed, they might also be due to the relative concentration of each TF in the various cell lines used in our assays rather than to the DNA affinity of each TF for the silencers.

Together, the profiles of TF binding sites in the *mariner* silencers looks like the numerous PRE/TRE that have previously been described in *D*. *melanogaster* and the few well-characterized PRE/TRE in mammal genomes [31,32]. Because the closest TSS upstream of these MLE silencers is that of their transposase ORF, these PRE/TRE were probably originally dedicated to the repression of the MLE transposon activity in cells expressing one or several of the identified TF candidates. Because MLEs have co-evolved with their animal hosts it is no surprise to observe that they have co-evolved to use conserved TFs and host housekeeping pathways to control their activity.

### Specificity of TFs involved in activity of *mariner* silencers

To our knowledge no functional link between NFAT-5, Alx1 and NRSF in adult insects or vertebrates have been published. Indeed, NFAT-5 is primarily implicated in the response to osmotic stress [58–60], Alx1 in osteogenesis during vertebrate development [61], and NRSF as a negative regulator of neuronal fate by silencing neuronal-specific genes in non-neuronal cells [62,63]. Furthermore; NFAT-5 and Alx1 appear to share with NRSF the property of participating at different levels in the development and differentiation of the nervous system [64–67]. NRSF was also reported to be involved in non-neuronal pathways of development or cell differentiation [67–69]. Even if the role of NRSF in the functioning of *mariner* silencers is not yet fully elucidated it suggests that the activity of these silencers in somatic cells might be dependent on the particular development pathway being used and the cellular environment. The fact that NRSF was involved in a polycomb dependent silencer is of interest. Indeed, this TF was found to have context dependent functions for the PRC1 and PRC2 recruitments [70–72] and is able to act as a recruiter for both complexes or as a limiting factor for the PRC2 recruitment [71]. NRSF is therefore an excellent candidate to positively or negatively regulate the commitment of *mariner* silencers in the polycomb pathway. Confirmation of a functional interplay between NRSF and MLEs would match well with the host range of these TEs. To our knowledge, MLEs are restricted to animal genomes having a nervous system (i.e. present in the genomes of cnidarians through arthropods and chordates). Nevertheless, it should be noted that in the original manuscript describing the discovery of *mariner* in *D*. *mauritiana* [73] *mariner* activity was not restricted to a particular cell type. Indeed, although the excision activity of *Mos1* from the white peach locus was found to occur in neuron-like primordial cells of eye facets, it also occurs in the primordial cells of the larval Malpighian tubules and adult male testis sheaths.

The silencing machineries used by *mariner* silencers can also explain why neo-integrated *Mos1* transposons are so stable and inefficient for remobilisation in transgenic insects [74]. Indeed, when silencing pathways promote and propagate H3K27 trimethylation in the neighbouring regions of their primary binding site [75], the MLE silencer element could extend the silent state of chromatin beyond the transposon, making it inaccessible for the transposase. Self-regulation using host silencing pathways is therefore potentially a mean to control MLE activity at two levels: transposase expression and transposon mobilization.

### TEs self-silencing by the host silencing machineries

In somatic cells TEs can either move or rearrange themselves within the genome. Therefore, they need to be finely tuned to avoid deleterious side effects due to their activity. Until now it was the TE host that was most often considered the main actors of this control or defence against TEs, using epigenetic mechanisms including RNA interference (RNAi), DNA methylation and histone modifications to silence TE transcriptional activity. In spite of their widespread presence in animal genomes, master loci coding for small interfering RNA and other host mechanisms have not, so far, been demonstrated to be an important mechanism for repression of MLE transcription in animal genomes [76,77]. It is therefore possible that other mechanisms exist that control MLE transcription. Our results support that, just as certain viruses and endogenous retroviruses [20–30], MLEs control their activity using a self-regulation mechanism that uses the host polycomb machinery and certain host TFs. This self-regulation would not be the only mechanism that is controlled by MLEs. Indeed, cells that temporarily do not express TFs that elicit *mariner* silencers also show evidence of self-repression. Two other non-exclusive mechanisms were proposed to also mediate MLE self-repression. Beyond a certain threshold of transposase concentration, the first mechanism would lead to a partial or complete transposase aggregation outside the nucleoplasm, the compartment in which MLE transposition occurs. This sequestration would likely depend on certain host proteins [78]. The second mechanism would rely on communication between transposase subunits, their concentration, and the number of transposons that can be mobilized in the environment [79]. Whatever the features of their hosts and the role of these mechanisms, it is striking that MLEs might use certain host housekeeping pathways as the main modulator of their expression. This also applies to MITE derivatives that lack a silencer (*e*.*g*. *MADE1* for *Hsmar1* [80]), but their mobility is controlled *via* availability in the nuclear environment of transposases encoded by related functional elements.

Overall, data accumulated on the self-management of some herpesviruses and retrovirus latency by using host silencing machineries support the suggestion that some endogenous retroviruses and MLEs are themselves the main actors of their “latency” regulation in the germ line and the soma of their hosts. This view is a breakthrough compared to the widely accepted idea that the host restrain the activities of all TEs in its genome. It also suggests that some TEs might be able to master their own invasion dynamic within their host genome, and that this would vary depending on their ability to use the host silencing machineries.

This change in the conception of TE activity does not modify our understanding of their involvement in the host genome evolution. It is tempting to propose that insertions of MLEs might have had beneficial effects for their host's evolution by spurring the complexity of silencing regulatory networks [6,81]. The presence of human MLEs within genic regions supports this hypothesis. However, their distribution might also reflect their preference for inserting into gene loci. This could, for example, be because they would be more accessible to the MLE insertion complex. Silencers are not only located in gene promoters, several of them are scattered downstream of the TSS and the stop codon [82]. As supported by our data, such locations would not hamper the ability of each of these elements from participating in fine-tuning gene expression based on developmental stage, tissue, and cell type. Further investigations will be necessary to develop efficient experimental approaches to determine whether MLE silencers i) use one or several host silencing pathways to be effective, ii) have a silencer activity that is fully ubiquitous in animal bodies or have an activity that can cease at some steps of the life cycle, and iii) were exapted several times in order to intervene in the silencing regulation networks during evolution of animal taxa [83,84].

### Impact of *mariner* silencers on the expression of their host genome

Although we indicated in the result section that the data of our *in silico* investigations must be viewed only as a prospective study, they suggest that part of the 109 *Hsmar2* silencers located in genic regions have kept their ability to induce the PRC2 silencing pathway. Taking into account the lack of transposition activity of *Hsmar2* in the human genome and their sequence degeneracy, the conservation of this ability to silence chromatin might have been exapted during evolution by the host genome. Therefore, it might correspond to a putative network of *Hsmar2* PRE-like interspersed in certain genic regions of the human genome.

Concerning *Hsmar1*, we were disappointed when we did not obtain a correlation similar to that obtained with *Hsmar2* silencers. Indeed, our experimental data in HeLa cells supported that at least a part of *Hsmar1* silencers efficiently silenced gene expression. However, our i*n silico* approaches failed to reveal an impact on local histones. This could be explained by two hypotheses. The first is that *Hsmar1* elements have so far not been exapted for this functionality in the human genome. Therefore, the status of their current chromatin was gradually dictated by their genomic environment throughout their evolutionary sequence inactivation. The second implies that only a small population would currently be exapted in the human genome, which hampers localizing them with our analytical approaches. Previous reports support this second hypothesis since a small part of TEs (~5%) located near genes undergo purifying selection in mammal genomes, and might have regulatory functions at the levels of histone modification or gene expression [56,85–87]. Novel approaches will also be necessary to investigate the possible role of *Hsmar1* and *Hsmar2* silencers and whether they were also exapted during human evolution.

As noted above, MLEs have co-evolved with their animal hosts and it is therefore not a surprise to observe that they use certain housekeeping proteins to control their activity. Even if our results do not elucidate the involvement of NRSF in the functioning of the *mariner* silencers and its possible links with Alx1 and NFAT-5, we found information in various databases and in the literature indicating that part of the genes containing a *mariner* silencer might be related to the functioning of neuron and the central nervous system. Unfortunately, these preliminary data were not statistically confirmed using facilities of the GREAT platform [88].

Future investigations will require the development of specific approaches to further scrutinize and confirm the determinants of the *mariner* silencers. Another important issue will be elucidating what the development, differentiation, or physiological pathways are, how they might intervene, and to confirm that they were exapted during evolution of the human genome, and/or in any other animal genomes in which they are widespread.

## Methods

### Cell lineages

Six cells lineages were used. Dr G. Sui (Harvard Medical School, ME USA) provided DT40 and DT40yy1- cells, Dr M. Esteller (CNIO, Madrid, Spain.) provided Co115 cells and Dr HY. Hwang (Standford University, USA) provided Speedy (known as 91.1.F1) cells. Sf21 cells were acquires from Sigma-Aldrich, HeLa-S3 and H4 cells from the ATCC.HeLa cells derived from human cervical cancer cells and H4 cells from malignant human glioma were cultured in DMEM (Gibco) supplemented with 10% fetal bovine serum (Gibco). DT40 cells from chicken B lymphoma were cultured in RPMI-Glutamine (Gibco) supplemented with 10% fetal bovine serum and 1% chicken serum (Gibco). The lineage of malignant human colorectal cells Co115 was cultured in RPMI-Glutamine supplemented with 10% fetal bovine serum. The *Xenopus tropicalis* speedy cell line [14] is a secondary lineage derived from a primary lineage established from a *X*. *tropicalis* limb. Cells were cultured in 67% (v/v) L15 medium adjusted to amphibian osmolarity by dilution with sterile water, supplemented with 10% heat inactivated fetal bovine serum (Sigma) and a cocktail of penicillin G (50U/mL) and streptomycin (50μg/mL) (Invitrogen). Sf21 cells from *Spodoptera frugiperda* ovary were cultured in Grace’s insect medium with L-glutamine (Gibco) supplemented with 10% fetal bovine serum.

### Plasmid constructs for stable expression assays

pBlueScript SK+ plasmids were used as a vector backbone to make constructs for the stable expression assays. A [*NeoR*] marker cassette corresponding to a neomycin resistance gene coding a neomycin phosphotransferase 2 was cloned between the *Eco*RI and *Bam*HI sites of pBS SK+. This gene was flanked by an early SV40 promoter (a moderate promoter) and an SV40 terminator except for the plasmids used in Sf21 cells, where the SV40 promoter was replaced by the immediate early protein 1 promoter (IE1; baculovirus AcMNPV). Each assayed DNA segment was cloned upstream (*Eco*RI site) or downstream (*Not*I site) of the marker in positive (+) or negative (-) orientation. DNA spacers of 1.2 kbp or 2.7 kbp were cloned between the 3’ end of the marker and the 5’end of the assayed DNA segment at the *Xba*I site as described [89].

### Stable expression assay

Cells were co-transfected with approximately 150 ng of a two plasmids mix using jetPEI^TM^ as described by the manufacturer (Polyplus Transfection). Two third of the mix (100 ng) corresponded to the pGL3 plasmid (Promega), used to check for effective transfection. One third (50 ng) consisted of the assayed DNA plasmid. The amount of plasmid was fitted to its size with respect to that of the smallest plasmid used as a control in each experiment, [*NeoR*]. Two days after transfection, 1/3 of the transfected cells were evaluated for luciferase activity with the Luciferase Assay System Kit (Promega). The remaining 2/3 of the cells were transferred in 100 mm Petri dishes followed by G418 sulfate selection (800 μg/mL, PAA France) for 15 days. Cells were then fixed and stained with 70% EtOH-0.5% methylene blue for 3 h. Only colonies with a diameter > 0.25 mm were counted.

### Transient luciferase expression assay

Plasmid constructs are presented in S2 Fig. The fragments pMos1, pHsmar1, Δ8-*MOS1*-Δ*NRSF*, Δ8-*MOS1*-^*mut*^*NRSF*, Δ8-*HSMAR1*-Δ*NRSF*, and Δ8-*HSMAR1*-^*mut*^*NRSF* were synthesized by ATG:Biosynthetics. To use the plasmids containing promoter pMos1 or pHsmar1 in transient luciferase expression assay, the *Nco*I-*Bam*HI DNA fragment containing the luciferase ORF and an SV40 late polyadenylation signal was purified from the P_Luc plasmid, then cloned into each of both plasmids between *Nco*I and *Bam*HI sites. In pMos- and pHsmar1-Luc plasmids, the *Bam*HI site at the 3’ end of the luciferase cassette was used to clone the DNA fragment to assay. For the transient luciferase expression assay in HeLa and H4 cells, 6 x 10^4^ cells were seeded onto a 24-well plate one day prior to transfection. Transfection was performed using jetPEI, according to the manufacturer’s instructions, using 400 ng of test DNA and 50 ng of pRL-Tk *Renilla*. For DT40 cells, 5 x 10^5^ cells were seeded onto a 24-well plate one day prior to transfection. jetPEI was also used to transfect about 400 ng of test DNA and 50 ng of pRL-Tk *Renilla*. For Co115, 4 x 10^5^ cells were seeded onto a 24-well plate one day prior transfection. For each test plasmid, its amount (400 ng) was fitted to its size with respect to that of the smallest plasmid used as a control in each experiment, P_Luc. Transfection was performed with ICAFectin441 DNA transfection reagent, according to the manufacturer’s instructions (In Cell Art), using 400 ng of test DNA and 400 ng of pRL-Tk *Renilla*. Luciferase expression was measured in a 96-well plate format with detection of fluorescence using the Dual-Glo Luciferase Assay System (Promega). Measurements were recorded on a Berthold plate-reader luminometer. Similar assays were used to investigate whether PRC2 was involved in the silencing effect observed with our constructs. However, a PRC2 inhibition was achieved by adding DZNEp (Sigma-Aldrich, USA) in the cell culture medium from the seeding until measuring *Firefly* and *Renilla* luciferase activities.

### Cell profiling

The expression profile of NRSF, YY1, TARBP2, Alx1, and NFAT-5 in HeLa, Co115 and H4 cells was determined by Western-blot analysis using commercial antibodies for NRSF (ab75785; Abcam), YY1 (ab12132; Abcam), TARBP2 (ab42018; Abcam), Alx1 (ABIN785202; antibodies-online GmbH) and NFAT-5 (ABIN183505; antibodies-online GmbH). For DT40 and HeLa cells, expression profile was determined by RNA-seq analysis using data available in databases, GEO datasets SRX286375 and SRX083286, respectively. During the analysis, we observed only one discrepancy between the RNA-seq data and the Western-blot analyses. Indeed, our HeLa cells were found to express *NFAT-5* whereas the RNA-seq analyses done on another HeLa cell batch led to the opposite conclusion.

### Transient GFP expression assay and flow cytometry analyses

8 x 10^4^ cells were seeded onto a 24-well plate one-day prior transfection and then transfected with jetPEI, according to the manufacturer’s instructions using 0.5 μg of plasmid DNA. Cells recovered from the culture 24 h post-transfection were washed three times with 1X PBS. The cell pellet was finally suspended in 400 μL 1X PBS-2% paraformaldehyde (w/v), and stored at 4°C. The analyses were performed using a flow cytometer FACSORT and the Cell Quest program (Beckton Dickinson). A total of 20 000 cells were acquired for each sample. Dead cells and debris were excluded from the analysis based on forward angle and side scatter light gating. Analysis gates were determined from the green fluorescence intensity using transfection controls done with or without plasmids expressing GFP.

### EMSA

#### NRSF proteins

Two plasmid expression systems that had been previously used in HeLa cells by the teams of Louis B. Hersh (University Kentucky, USA) and Wei-Ping Yu (National Neuroscience Institute, Singapore) were used here as well. The first system expresses pFLAG-human *NRSF*, a human NRSF fused at its N-terminal end with a FLAG antigen [28]. The second, pCMVß-*myc*-fugu-*NRSF*/*REST*, expresses a fugu NRSF fused at its N-terminal end with a myc antigene [29]. The FLAG and myc tags allowed us to follow the transient expression of the NRSF proteins by Western-blot analysis with specific anti-FLAG or -myc antibodies (Sigma and Abcam, respectively). They also allowed confirmation that the shifted bands observed in EMSA are specific NRSF/binding site complexes by performing super-shift assays with appropriate anti-tag monoclonal antibodies. A third expression system was set-up to express the drosophila version of NRSF, charlatan (chn), in HeLa cells. The *chn* ORF was recovered from Addgene (plasmid # 39679). The fragment *Psp*OMI-*Eco*RV containing the *chn* ORF was cloned into the mammalian expression plasmid pVAX1, between *Not*I and *Xba*I cleavage sites, after filling of the *Xba*I end with the Klenow DNA polymerase.

#### Preparation of HeLa cell nuclear extracts (NE)

1 x 10^7^ HeLa cells were plated in Petri dishes (Ø = 10 cm) one-day prior transfection and transfected with 15 μg of each plasmid expressing an NRSF using jetPEI according to standard protocols. One day post-transfection the cells were scraped off and washed with PBS 1X solution. NEs were prepared by high salt extraction method. Briefly, HeLa cells transfected with pFLAG-human *NRSF*, pCMVßmyc-*NRSF*, pCMVß-myc-fugu-*NRSF/REST* or the mock plasmid (pCS2+) were lysed in sucrose buffer (0.32 M sucrose, 10 mM Tris–HCl pH 8.0, 3 mM CaCl2, 2 mM MgOAc2, 0.1 mM EDTA, 0.5% NP-40, 1 mM DTT and 1X Protease inhibitor cocktail [PIC; Sigma]) in 100 μL per 10^7^ cells. After gently mixing by pipetting, nuclei were collected by centrifugation at 500 g for 5 min at 4°C and resuspended in a low salt hypotonic buffer containing 20 mM HEPES (pH 7.9), 1.5 mM MgCl2, 20 mM KCl, 0.2 mM EDTA, 0.5 mM DTT, 1X PIC and 25% glycerol. The nucleoplasm was extracted by slowly adding an equal volume of a high salt buffer (20 mM HEPES pH 7.9, 1.5 mM MgCl2, 0.8 M KCl, 0.2 mM EDTA, 0.5 mM DTT, 1X PIC, 1% NP-40 and 25% glycerol). After rotating at 4°C for 45 min NEs were collected by centrifugation at 14 000 g for 15 min at 4°C. They were desalted using ZebaTM spin desalting columns (7KMWCO), as recommended (Thermo Scientific) and conserved at -80°C in 10 mM Tris–HCl pH 8.0, 150 mM KCl, 0.5 mM EDTA, 0.2 mM DTT, 0.1 mg/mL polydI-dC, 0.1% Triton X-100, 1X PIC, 12.5% glycerol. NE protein concentration was determined by a Bradford assay using bovine serum albumin as standard.

#### Probes

Probes were primer pairs synthesized by PCR. The first one was labelled with the ATTO fluorochrome (Eurogentec SA) carried by one of the primers, allowing direct detection of complexes in EMSA. The second probe was unlabeled and used as a competitor. PCR were monitored for 30 cycles (95°C 15 s, 60°C 1 min, and 72°C 30 s) from 20 ng of DNA plasmid as a template and GoTaq polymerase, under conditions recommended by the supplier (Promega). Each probe was then separated by electrophoresis onto a 2% Nusieve GTG agarose gel, purified by gel elution using a QIAquick PCR purification kit (Qiagen) and quantified with a BioSpec-nano (Shimadzu Biotech).

#### Binding reactions, gel electrophoresis and analysis

The binding reaction was performed by mixing 10 μg of NE with 0.02 pmole of ATTO-RE1 probe or 0.02 to 0.2 pmole of ATTO-Δ8 probe in 20 μL binding buffer (final concentration: 10 mM Tris–HCl pH 8.0, 150 mM KCl, 0.5 mM EDTA, 0.2 mM DTT, 0.1 mg/mL polydI-dC, 0.1% Triton X-100, 12.5% glycerol, 1X PIC) on ice for 1 h. For the super-shift assay, 1 μg of anti-FLAG or anti-myc antibody was added to the reaction mixture. For the competition assay, an excess of 100 to 200-fold of unlabelled probe was incubated with the NE on ice for 1 h. The ATTO labelled probe was then added in a final volume of 20 μL and incubated on ice for 1 h. Samples were separated by non-denaturing PAGE at 200 V and shifted bands were detected by near-infrared fluorometry, using an Odyssey Scanner (LiCor).

### CHIP-seq peak analyses

Annotations from RepeatMasker were used to select positions of *Hsmar1* and *Hsmar2* in the hg19 human genome version. Those containing a Δ8 segment (Sil+), a damaged Δ8 segment (U), or no Δ8 segment (Sil-) and their location in a genic or an intergenic region were inventoried using home made perl scripts (S1 Table). Here, genic regions corresponded to those for which maximal efficiency of the *mariner* silencer was observed from our experimental data (i.e. from the TSS of each gene to 5 kbp downstream of its 3’end). Intergenic regions corresponded to any region of the genome that was not genic. ChIP-seq peaks files (EzH2, H3K27me3, H3K27ac, H3K4me3, and H3K9me3; no data about H4K20me3 were available for all cell lines) were located within UCSC resources for ENCODE data and downloaded at https://genome.ucsc.edu/ENCODE/dataMatrix/encodeDataMatrixHuman.html [86]. Intersections between the location of ChIP-seq peaks and Sil+, Sil- or U elements were performed using bioconductor. The chromatin status of each silencer was then inventoried and classified in four main categories: (i) undetermined chromatin (ucs) when no peak co-localized, (ii) Su(var)39/HP1 (H) when H3K9me3 peaks co-localized, trithorax (T) when H3K27ac and-or H3K4me3 peaks co-localized, and polycomb when EZH2 and-or H3K27me3 peaks co-localized. Mixed statuses (T-H, P/H, P/T, P/T/H) were proposed when appropriate (S1 and S2 Tables). 96.7% (1792/1855) of the Sil+, Sil- and U elements had at least one annotation about their chromatin status. Consequently, we considered that “ucs” annotations were not due to weak mapping ability of *Hsmar1* and *Hsmar2* elements but rather to variation of the quality of the CHIP-seq signal probably because sequencing depths were not deep enough [90,91] and-or variation of “sequencing ability” from one locus to another [92–94]. Therefore, statistical analyses were performed using datasets in which polycomb, trithorax and Su(var)39/HP1 frequencies were calculated and differences between MLEs Sil+/Sil- was tested using a Wilcoxon signed-rank test without “ucs” data (S2 Table).

### Detection of YY1 and NFAT-5 binding sites in genomic *Hsmar2*

Using HMMER, a HMM model for YY1 was calculated from the YY1 binding sites found in *Mos1* and the consensus sequences of *Hsmar1*, *Hsmar2*, *Himar1* and *Mcmar2*. YY1 sites were then detected in genomic *Hsmar2* sequences using HMMER and score for positive hits were recorded. Textual searches were done to identify putative NFAT-5 binding site using the motif AAGGG/CCCTT.

### Statistics

The graphics calculated from data analysed with non-parametric statistics followed recommendations of the guidelines for journals of the American Society of Microbiology [95]. All values represented in graphics corresponded to the median value obtained from three experiments done in triplicate (9 data points). Bars corresponded to the values of quartiles 1 and 3. In the text, figures and supplementary data, all the results indicated as being different were previously verified to be significant with a p-value < 0.05, using a Kruskal-Wallis test. Wilcoxon signed-rank tests, Student t-tests and hierarchical clustering were done using R facilities and libraries [96].

## Supporting Information

S1 Fig Principle of the transient luciferase expression assay. To study the effect of transient expression of a DNA segment, the assay system for detection and characterization of silencer and enhancer-blockers (EB) set up by Dr L. Elnitski’s team was used [13]. Briefly, this system is based on the transient expression of two plasmids. The first of these is the pRL-Tk plasmid that expresses the *Renilla* luciferase under control of a Thymidine kinase promoter. Its transient expression is followed as a control for transfection efficiency. The second plasmid is a derivative of the pGL3 plasmid that expresses the *Firefly* luciferase under control of an early SV40 promoter. Features of the pGL3 plasmid derivatives are shown in the figure above and described next. P_Luc and HS2_P_Luc plasmid are used as expression controls to identify the thresholds that allow characterization of the DNA element under investigation (EUI). P_Luc is a pGL3 plasmid. HS2_P_Luc is a pGL3 plasmid in which the core human beta-globin HS2 enhancer has been cloned upstream of the pSV40 promoter. In the HS2_P_Luc plasmid a *Psp*OM1 site is present upstream of the HS2 enhancer, as well as a *Bgl*II site between the HS2 enhancer and the pSV40 promoter and a *Bam*HI site downstream of the luciferase gene terminator. These three restriction sites can be used to clone the DNA EUI in the plus or minus orientations. Schematic representation adapted from [13].
(DOCX)

S2 Fig Impact of spacer length on the silencer effect of the Δ7-*MOS1* segment when linear DNA vectors are used in a stable gene expression assay. We assayed the impact of plasmid configuration using linear vectors in which the [*NeoR*] cassette was separated at its 3’ end by a 3 kbp DNA fragment from a Δ7-*MOS1* segment (in positive or negative orientation). The distance effect was analysed by transfecting these linear vectors into HeLa cells and screening for neomycin resistant colonies after 2 weeks of G418 selection. Each histogram bar corresponds to the median value obtained from three experiments performed in triplicate. Bars corresponded to quartiles 1 and 3. * indicates significant differences (p<0.05) compared to the [*NeoR*] control. Results indicate that a Δ7-*MOS1* segment located 3 kbp downstream of the *NeoR* marker gene is able (with statistical significance) to negatively interfere with its expression only when it is located in a positive orientation. Overall, these results support the conclusion that the Δ7-*MOS1* segment has its optimal silencer effect when it is located downstream of the maker gene in a positive orientation. For these experiments the linear vectors were prepared as follows: first, pBS plasmids containing the [*NeoR*], [*NeoR*]Δ7+ or [*NeoR*]Δ7- were linearized by enzymatic cleavage using *Eco*RI. The linear vectors were purified by electrophoretic separation on agarose gels, eluted with a QIAquick PCR purification kit (Qiagen), and quantified with a BioSpec-nano (Shimadzu Biotech).
(DOCX)

S3 Fig DNA sequences of the *Mos1* and *Hsmar1* promoters. The design of both promoter sequences took into account the definition previously proposed for *Hsmar1* [16]. The 5’ ITRs are shown inside the boxes and are flanked at the 5’ end by a TA dinucleotide that is duplicated during insertion, and at the 3’ end by the complete 5’ UTR that ends just before the ATG codon of the transposase ORF. The multi-cloning site (MCS) at the 3’ end (shown in blue and red) can be cleaved by *Nco*I, *Bcl*I, *Sal*I, *Bam*HI, *Eco*RI, and *Bgl*II. The *Nco*I and *Bam*HI sites of this MCS were used to clone an *Nco*I-*Bam*HI DNA fragment (1924 bp) containing an ORF coding the firefly luciferase, which was purified from a pGL3 plasmid (P_Luc). pMos1 and pHsmar1 DNA fragments were synthesized by ATG:biosynthetics GMBh (Merzhausen, Germany) and each cloned in a pUC19 plasmid.
(DOCX)

S4 Fig Features of the Δ8-*MOS1* segment and its variants. (**a**) Location of the Δ81 to Δ85 *MOS1* DNA segments within the sequence of the Δ8-*MOS1* segment. Δ8, black; Δ81, red; Δ82, blue; Δ83, green; Δ84, purple; Δ85, orange. The region absent in the short version of Δ81 (short Δ81 probe in Fig 7F) is highlighted in grey in the sequence of Δ81. Transcription factor binding sites frequently found within PREs in drosophila are shown and are highlighted in blue for YY1 or Pho, green for Ezh2 or Zeste, turquoise and pink for the GAGA and GTGT factors, respectively. Other binding sites are in red for NRSF, grey for NFAT-5, and black for Alx1. (**b**) Comparison of effects of Δ7-*MOS1* and Δ8-*MOS1* on the expression of the *Firefly* and the *Renilla* luciferase marker genes using transient expression assays in HeLa cells. The assays were performed with Δ7 and Δ8 segments cloned in + orientation. Each histogram bar corresponds to the median value obtained from three experiments done in triplicate. Bars corresponded to quartiles 1 and 3. The median ratios RLU from *Firefly*/RLU from *Renilla* were calculated as indicated in Fig 2. The area where the ratios “RLU from *Firefly*/RLU from *Renilla*” were above 1 (i.e. where no strong silencer effect is observed) is coloured in grey. No significant effect was found between both plasmid constructs (p<0.05).
(DOCX)

S5 Fig Putative binding sites of TFs within Δ7 segments of *Himar1*, *Mcmar1*, *Hsmar1*, and *Hsmar2*. Black: Δ8 *mariner* segments; black+grey: Δ7 *mariner* segments. Transcription factor binding sites frequently found within PREs in drosophila are shown in blue for YY1, in green for Zeste, in turquoise for the GAGA factor and in pink for the GTGT factors. Arrows above the nucleic acid sequences indicate the orientation of each motif. NRSF binding sites are highlighted in red and typed in white. With respect to NRSF binding sites that are conserved in position in all MLEs, the other motifs are arranged in different configurations, ordering and spacing in each element. This indicated that these motifs would not have been conserved in orthologous positions across MLEs during their evolution. Putative NFAT-5 binding sites are highlighted in boxes.
(DOCX)

S6 Fig Sequence conservation of the NRSF binding site among *mariner* nucleic acid sequences. Conserved motifs were searched within the nucleic acid sequences corresponding to the Δ7 DNA segment of 34 *mariner* elements using the MEME facilities at http://meme.sdsc.edu/meme/cgi-bin/meme.cgi. A single conserved motif of 50 nucleotides was found and was located in the region encoding one of the two highly conserved peptide motifs in the *mariner* transposase, PHxxYSPDLAPxD [34]. We found that this conserved 50 bp motif also contained a 29 bp NRSE with a 10 bp spacer between both conserved moieties, rather than a 2 bp spacer as found in the cardinal NRSF binding site (RE1). Charlatan is the ortholog of NRSF in diptera. The names of each of the 34 *mariner* elements are shown on the right side of the figure. Their names, accession numbers and host species for each element are: Tvmar1, AY282463 *Trichomonas vaginalis*; Ahmar1, AB056896 *Adoxophyes honmai*; Armar1, AB056894 Ascogaster reticulatus; Cpmar1, U11641 *Chrysoperla plorabunda*; Mpmar1, U11649 *Mantispa pulchella*; Damar1, U11656 *Drosophila ananassae*; Himar1, U11646 *Haematobia irritans*; Hsmar2, U49974 *Homo sapiens*; Bytmar1, AJ507226 *Bythogrea thermydron*; Acmar1, AB081476 *Apis cerena*; Ammar1, U19902 for *Apis mellifera*; Ccmar1, U40493 *Ceratitis capitata*; Demar1, U08094 *Drosophila erecta*; Famar1, AY226507 *Forficula auricularia*; Gpmar1, U18308 *Glossina palpalis*; Aamar1, AB006464 *Attacus atlas*; Dtmar1, X79719 *Dugesia tigrina*; Funmar1, AB055188 Fungia sp. Kusabiraishi; Hbmar1, U04455 *Heterorhabditis bacteriophaga*; Hcmar1, M63844 *Hyalophora cecropia*; Hsmar1, U52077 *Homo sapiens*; Cemar1, M98552 *Caenorhabditis elegans*; Cemar2, X77804 *C*. *elegans*; Mcmar1, H20772 *Meloidogyne chitwoodi*; Bcmar1, AF349133 *Bactrocera tryoni*; Botmar1, consensus sequence (personnal data) *Bombus terrestris*; Dsecmar1, AF035569 *Drosophila sechellia*; Madmar1, U24436 *Mayetiola destructor*; Mbmar1, AF465247 *Mamestra brassicae*; Mlmar1, AC182003 (element from 70120 to 71051) *Myotis lucifugus*; Dmmar1 = *Mos1*, X78906 *Drosophila mauritiana*; Momar1, U12279 *Metaseuilius occidentalis*; Mudmar1, AF373028 *Musca domestica*; Sinvmar1, AF518173 *Solenopsis invicta*. Depending on the subfamily to which they belong, the element names are typed in brown (*irritans)*, green (*capitata/mellifera)*, blue *(cecropia)*, purple (*briggsae/elegans)* and red *(mauritiana)*. Tvmar1 is the only member of what might be the sixth *mariner* subfamily. The PHPPYSPDLAPXD motif is given above the sequence alignment. Consensus of NRSE [35, 36] (so called RE1) and CBE [37] (*charlatan* binding element) are indicated below the sequence alignment. In the consensus, the most conserved positions are shown in upper case and the variable positions in lower case. In the CBE consensus, IUPAC abbreviations are used to indicate the following: S = C or G; K = G or T; B = C, T or G; H = A, T or C; M = A or C; V = A, C or G and N = A, C, G or T. Taking into account the sequence degeneracy of the CBE motif, the positions that are conserved in the binding motif are highlighted in grey.
(DOCX)

S7 Fig Set-up of the EMSA for validating the use of nuclear extracts containing the drosophilan NRSF (chn), the human NRSF (HsNRSF) or the fugu NRSF (fuguNRSF) proteins. **(a)** The RE1 probe corresponds to the RE1 binding site located upstream of the vesicular acethylcholine (VAChT) transporter and choline acethyltransferase (ChAT) genes in the human cholinergic gene locus. A 120 bp DNA segment was amplified by PCR from the pKL-7 plasmid [38]. Primers are highlighted in grey and the RE1 site in yellow. ATTO fluorochrome located at the outer 3’ end in the labelled probe is shown in bold. **(b)** Set-up of the EMSA for chn. Lane 1 corresponds to a probe control. Lanes 2 to 4 were obtained with a nuclear extract (NE) containing chn. Because chn was not tagged with a peptide and we did not have anti-chn antibodies, the specificity of the shifted complex observed in lane 2 was verified in lanes 3 and 4 by observing binding with a specific DNA binding competitor (CBE-1: 5’-GGCCGTTCAGCACCACCGCCATTGGTCGCGC-3’ [37]) and a non-specific competitor (MCS; sequence in S8 Fig), as described previously [37–38]. **(c)** Set-up of the EMSA for HsNRSF and fuguNRSF. Lanes 1 and 10 correspond to probe controls. Lanes 2 through 5 and 11 through 14 are negative controls obtained with an NE prepared from HeLa cells transfected with an empty expression plasmid: pCS2+. Lanes 6 through 9 were obtained with an NE containing HsNRSF fused to a FLAG tag. The specificity of the shifted complex observed in lane 6 was verified in lane 8 in which the anti-FLAG monoclonal antibody (Anti-FLAG MAb) creates a super-shifted complex (red stars). Lanes 15 to 18 were obtained with an NE containing fuguNRSF fused to a myc tag. The specificity of the shifted complex observed in lane 15 was verified in lane 16 in which the anti-myc monoclonal antibody (Anti-myc MAb) creates a super-shifted complex. Lanes 7, 9, 17 and 18 were negative controls that support the conclusion that the super-shifted complexes observed in lanes 8 and 16 were not due to a non-specific effect of the MAb. Complexes were separated on a 6% PAGE in 0.25X TBE gel. Together, these results support that the three NRSF systems can be used to detect NRSF binding sites with EMSA.
(DOCX)

S8 Fig **Location of the variant segments within the sequences of the Δ8-*MOS1* (a) and Δ8-*HSMAR1* (b).** Names of each variant are indicated at the 3’ end of its sequence. Dashes indicated positions that are present in each fragment. Conserved DNA binding motifs described in Fig 6B are highlighted in red for NRSF, blue for YY1 or Pho, green for Ezh2 or Zeste, turquoise and pink for the GAGA and GTGT factors, grey for NFAT-5, and black for Alx1, respectively. (**c**) Expression of the *Firefly* and the *Renilla* luciferase marker genes using transient expression assays in HeLa cells. The assays were performed with Δ8-*HSMAR1* and five variants cloned in + orientation. Each bar corresponds to the median value obtained from three experiments done in triplicate. Bars corresponded to quartiles 1 and 3. The median ratios RLU from *Firefly*/RLU from *Renilla* were calculated as indicated in Fig 2. The area where the ratios “RLU from *Firefly*/RLU from *Renilla*” were above 1 (i.e. where no strong silencer effect is observed) is coloured in grey. * indicates a significant difference (p<0.05) with the P_Luc controls. ** indicates a significant difference (p<0.05) with HS2_P_Luc_Δ8-*HSMAR1*.
(DOCX)

S9 Fig DNA binding of fuguNRSF to a shortest version of the Δ81 segment of *MOS1* (10 pmole of ATTO-labeled probe/lane; Sequence supplied in S4 Fig). Lanes 1 and 4 correspond to probe controls. Lanes 2 and 5 show shifted complexes with NE containing fuguNRSF. The grey arrow locates a complex resulting from the binding of YY1, as shown in Fig 6D. The specificity of this YY1 complex in lanes 2 and 5 was verified by adding 10X of unlabelled Δ84 DNA segment that contains two YY1 binding sites. The red stars in lanes 2 and 5 indicate a complex that is absent when HeLa NE are used (Fig 6D). The involvement of fuguNRSF in this second complex is shown in lane 6, using a specific antibody that leads to its destabilization. It is noticeable that this second complex is also sensitive to the competition with the unlabelled Δ84 DNA segment (lane 3).
(DOCX)

S10 Fig Effect of a DZNep treatment on the functioning of Δ8-HSMAR1 variants. Expression of the *Firefly* luciferase gene from 4 different HS2_P_Luc plasmid constructs containing Δ8-*HSMAR1* variants cloned downstream of the marker gene. Each histogram bar corresponds to the median value obtained from three experiments done in triplicate. Bars corresponded to quartiles 1 and 3. * indicates a significant difference (p<0.05) between cells treated or not with 5 μM DZNep. As indicated in Fig 9, the ratio “RLU from *Firefly*/RLU from *Renilla*” for the HS2_P_Luc transfection done in absence or presence of 5 μM DZNep are used as a reference and fixed at 1 arbitrary unit. They were respectively used to calculate the ratios of each construct assayed in absence or in presence of 5 μM DZNep.
(DOCX)

S11 Fig Sequence features of *Hsmar1* and *Hsmar2* elements in the human genome. (**a**) *mariner* elements and Δ8 *mariner* segments were counted using the RepeatMasker annotation in the Hg19 version. (*) number of elements with a maximum of 40 bp truncated at their 5’ and-or 3’ ends. Damaged Δ8 *mariner* segments are DNA regions containing one or several DNA insertions or deletions. (**b**) Conservation profiles in terms of sequence divergence and integrity of *Hsmar1* and *Hsmar2* elements in the human genome. The horizontal axes are calibrated to the size of the *mariner* elements. The vertical axis represents the number of stacked *mariner* elements. Each horizontal lane corresponds to one *mariner* element that consists in 1 to 6 high-scoring segment pairs (HSP). For *Hsmar1*, 469 elements are in 1 HSP, 106 in 2 HSPs, 16 in 3 HSPs, and 1 in 1 HSP. For *Hsmar2*, 838 elements are in 1 HSP, 280 in 2 HSPs, 6, in 3 HSPs, 28 in 1 HSP, 8 in 5 HSPs, and 3 in 6 HSPs. *Mariner* elements were ranked in the stacking from the best RepeatMasker scores to the worst. In the right margin a coloured scale from red to blue is shown, representing the sequence divergence of each element with its reference element (*Hsmar1*, AccN° HSU52077; *Hsmar2*; Acc N° HSU49974). Black bars above the horizontal axis show the location of the Δ8 segments.
(DOCX)

S12 Fig Metaprofiles of CHIP-seq signals and peaks in Sil+. Four loci containing a Sil+ *Hsmar2* inserted in positive orientation into an intragenic region and flanked by its 5' and 3' regions (each 2-kbp) were investigated in four cell lines (Dnd41, GM12878, HepG2 and HMEC) of ENCODE. *Hsmar2* Sil+ were those located into LOC 389996 (chr2:91,768,034–91,773,184) that overlaps with the otopetrin 1 pseudogene, and into intronic regions of the SLC22A16 (chr6:110,759,672–110,765,259), MYO5B (chr18:47,716,065–47,721,303), and PDS5A (chr4:39,833,821–39,838,393) genes. Graphics were calculated with Integrative Genomic Viewer (IGV2.3.63) and ENCODE data. On the top were supplied the locus name, a Refseq graphic of each locus, and the location of each *Hsmar2* element. Below, for each locus and in each cell line, the five first lanes described the CHIP-seq signal (named criterion-S on the right hand) for H3K27me3, EZH2, H3K4me3, H3K27ac and H3K9me3, respectively. The area in grey located absences of CHIP-seq signal. The signal scales at each locus were 60, 25, 25 and 25 in Dnd41; 50, 10, 25 and 7 in GM12878; 100, 20, 100 and 8 in HepG2; and 30, 15, 10 and 15 in HMEC. Results highlighted that the CHIP-seq signals varied importantly depending on the locus and the cell line. The five last lanes described the location of the CHIP-seq peaks (named criterion-P on the right hand; i.e. the statistically significant CHIP-seq signal calculated by ENCODE with a peak calling program subtracting the local signal input) for H3K27me3, EZH2, H3K4me3, H3K27ac and H3K9me3. The chromatin status of each locus in each cell type was indicated below between bracket with P, T, and H indicating a polycomb, trithorax or Su(var)39/HP1 status, respectively. “ucs”indicated an absence of co-localized peaks that was considered under our analysis conditions as an undetermined chromatin status. The 4 graphs at the bottom described the input signal for each locus in each of the four cell lines. The signal scales were 30 for LOC 389996, 15.36 for SLC22A16, 10 for MYO5B, and 15 for PDS5A. Results highlighted that the statistical significance of CHIP-seq signals through CHIP-seq peaks depended importantly on the input signal, locus and cell line. The blue band indicated the location of Sil+.
(PDF)

S13 Fig Heat map of the chromatin status of 187 intragenic *Hsmar2* silencers. The hierarchical clustering was performed analysing the chromatin status (P, T, H or ucs) of 187 loci. “+” in the right margin located the Sil+. Loci with a “ucs” chromatin phenotype were filled in grey. Loci mainly associated with a polycomb or a Su(var)39/HP1 status in the cell lines are gathered in the green and yellow boxes, respectively. Among loci with a polycomb status, those displaying a bivalent trithorax or Su(var)39/HP1 status were respectively in the blue box or in the orange boxes. 2A red-blue colour scale depicted normalized chromatin status (red: positive, white undefined, blue: absent). At the bottom of the heat map, the cell lines analysed for their polycomb, Trithorax or Su(var)39/HP1 status were respectively typed in green, blue or orange. The names referencing H1-ESC annotations were typed in purple.
(PDF)

S1 Table Inventories of putative silencers contained in *Hsmar1* and *Hsmar2*. (XLSX)

S2 Table Counts of the CHIP-seq peaks co-localizing with *Hsmar1* and *Hsmar2*. (XLSX)

S3 Table Chromatin status of the 8 *Hsmar1* Δ7 silencers in 14 human cell lines. (DOCX)
