# Supplementary figures and images for: Mariner Transposons Contain a Silencer: Possible Role of the Polycomb Repressive Complex 2
Source: PLoS Genet. 2016 Mar 3;12(3):e1005902. doi: 10.1371/journal.pgen.1005902 (PMC4777549; doi:10.1371/journal.pgen.1005902)

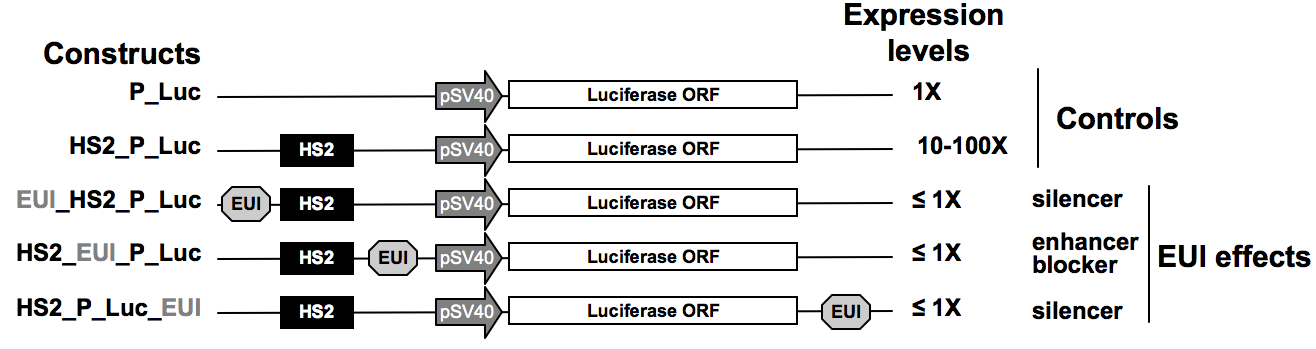

Supplement: S1 Fig — To study the effect of transient expression of a DNA segment, the assay system for detection and characterization of silencer and enhancer-blockers (EB) set up by Dr L. Elnitski’s team was used [13]. Briefly, this system is based on the transient expression of two plasmids. The first of these is the pRL-Tk plasmid that expresses the Renilla luciferase under control of a Thymidine kinase promoter. Its transient expression is followed as a control for transfection efficiency. The second plasmid is a derivative of the pGL3 plasmid that expresses the Firefly luciferase under control of an early SV40 promoter. Features of the pGL3 plasmid derivatives are shown in the figure above and described next. P_Luc and HS2_P_Luc plasmid are used as expression controls to identify the thresholds that allow characterization of the DNA element under investigation (EUI). P_Luc is a pGL3 plasmid. HS2_P_Luc is a pGL3 plasmid in which the core human beta-globin HS2 enhancer has been cloned upstream of the pSV40 promoter. In the HS2_P_Luc plasmid a PspOM1 site is present upstream of the HS2 enhancer, as well as a BglII site between the HS2 enhancer and the pSV40 promoter and a BamHI site downstream of the luciferase gene terminator. These three restriction sites can be used to clone the DNA EUI in the plus or minus orientations. Schematic representation adapted from [13]. (DOCX) [file pgen.1005902.s001.docx]

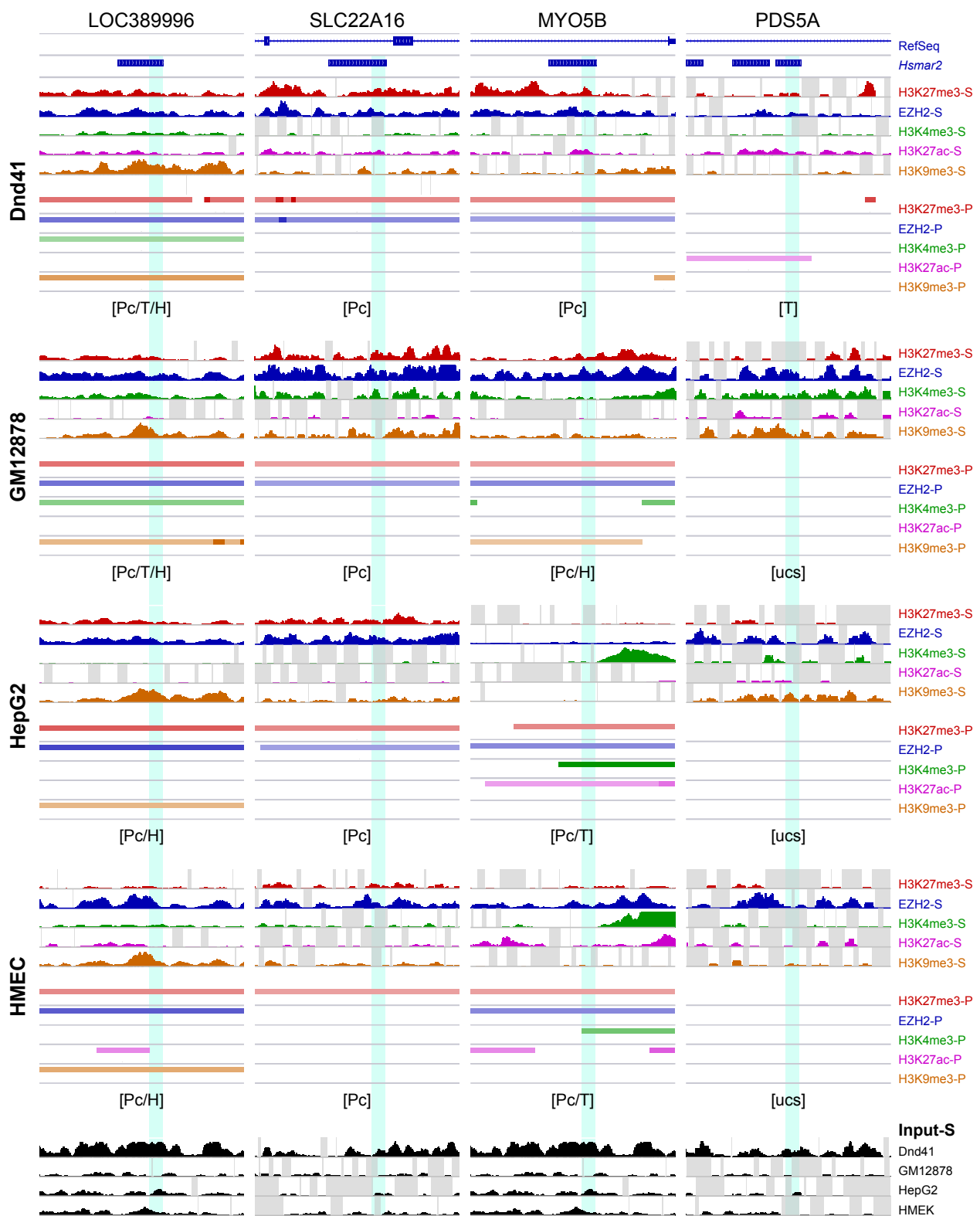

Figure S12

Supplement: S12 Fig — Four loci containing a Sil+ Hsmar2 inserted in positive orientation into an intragenic region and flanked by its 5' and 3' regions (each 2-kbp) were investigated in four cell lines (Dnd41, GM12878, HepG2 and HMEC) of ENCODE. Hsmar2 Sil+ were those located into LOC 389996 (chr2:91,768,034–91,773,184) that overlaps with the otopetrin 1 pseudogene, and into intronic regions of the SLC22A16 (chr6:110,759,672–110,765,259), MYO5B (chr18:47,716,065–47,721,303), and PDS5A (chr4:39,833,821–39,838,393) genes. Graphics were calculated with Integrative Genomic Viewer (IGV2.3.63) and ENCODE data. On the top were supplied the locus name, a Refseq graphic of each locus, and the location of each Hsmar2 element. Below, for each locus and in each cell line, the five first lanes described the CHIP-seq signal (named criterion-S on the right hand) for H3K27me3, EZH2, H3K4me3, H3K27ac and H3K9me3, respectively. The area in grey located absences of CHIP-seq signal. The signal scales at each locus were 60, 25, 25 and 25 in Dnd41; 50, 10, 25 and 7 in GM12878; 100, 20, 100 and 8 in HepG2; and 30, 15, 10 and 15 in HMEC. Results highlighted that the CHIP-seq signals varied importantly depending on the locus and the cell line. The five last lanes described the location of the CHIP-seq peaks (named criterion-P on the right hand; i.e. the statistically significant CHIP-seq signal calculated by ENCODE with a peak calling program subtracting the local signal input) for H3K27me3, EZH2, H3K4me3, H3K27ac and H3K9me3. The chromatin status of each locus in each cell type was indicated below between bracket with P, T, and H indicating a polycomb, trithorax or Su(var)39/HP1 status, respectively. “ucs”indicated an absence of co-localized peaks that was considered under our analysis conditions as an undetermined chromatin status. The 4 graphs at the bottom described the input signal for each locus in each of the four cell lines. The signal scales were 30 for LOC 389996, 15.36 for SLC22A16, 10 for MYO5B, and [file pgen.1005902.s012.pdf]

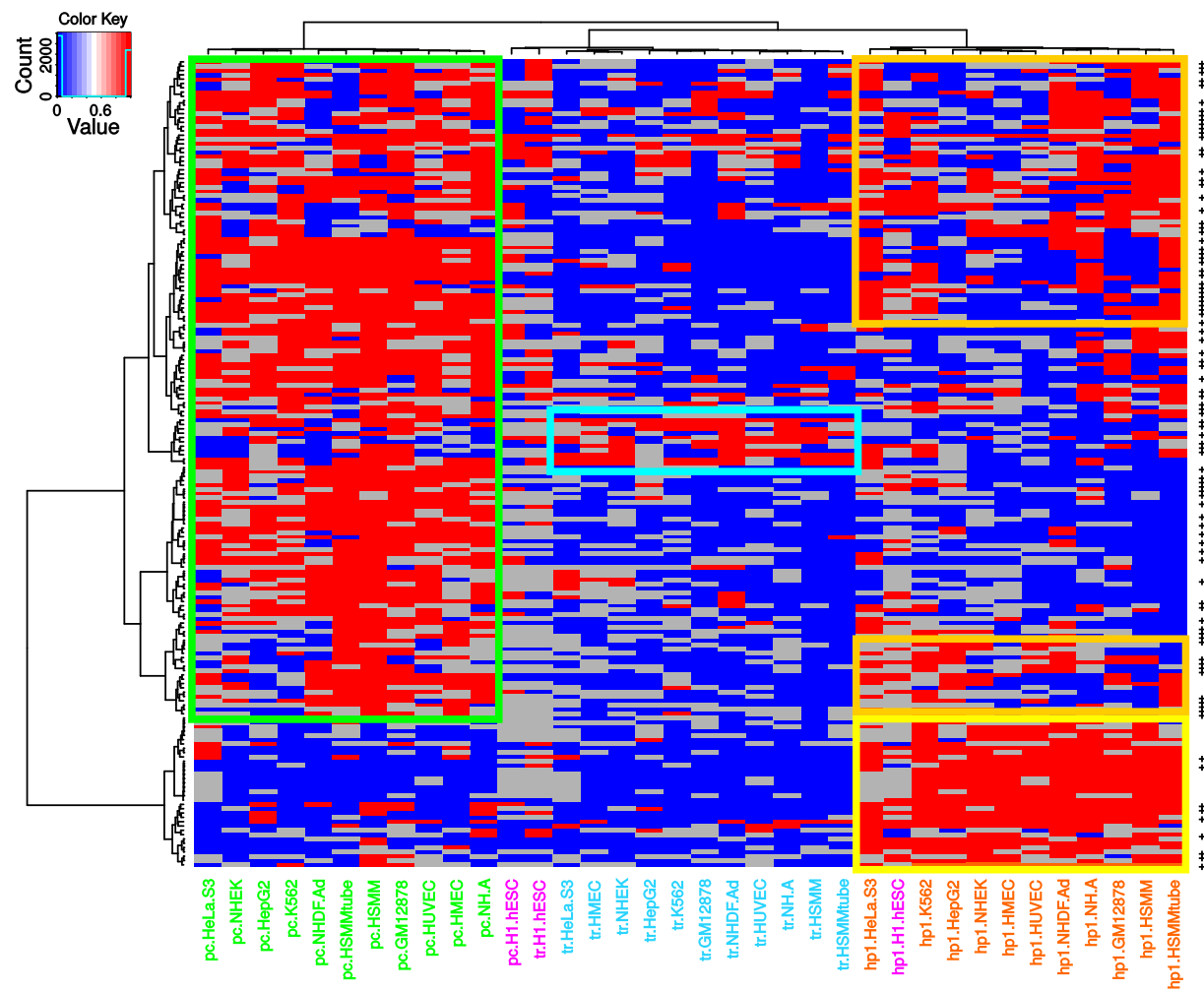

Figure S13

Supplement: S13 Fig — The hierarchical clustering was performed analysing the chromatin status (P, T, H or ucs) of 187 loci. “+” in the right margin located the Sil+. Loci with a “ucs” chromatin phenotype were filled in grey. Loci mainly associated with a polycomb or a Su(var)39/HP1 status in the cell lines are gathered in the green and yellow boxes, respectively. Among loci with a polycomb status, those displaying a bivalent trithorax or Su(var)39/HP1 status were respectively in the blue box or in the orange boxes. 2A red-blue colour scale depicted normalized chromatin status (red: positive, white undefined, blue: absent). At the bottom of the heat map, the cell lines analysed for their polycomb, Trithorax or Su(var)39/HP1 status were respectively typed in green, blue or orange. The names referencing H1-ESC annotations were typed in purple. (PDF) [file pgen.1005902.s013.pdf]
