# Supplementary material for: Mariner Transposons Contain a Silencer: Possible Role of the Polycomb Repressive Complex 2
Source: PLoS Genet. 2016 Mar 3;12(3):e1005902. doi: 10.1371/journal.pgen.1005902 (PMC4777549; doi:10.1371/journal.pgen.1005902)
Supplement: S3 Fig — The design of both promoter sequences took into account the definition previously proposed for Hsmar1 [16]. The 5’ ITRs are shown inside the boxes and are flanked at the 5’ end by a TA dinucleotide that is duplicated during insertion, and at the 3’ end by the complete 5’ UTR that ends just before the ATG codon of the transposase ORF. The multi-cloning site (MCS) at the 3’ end (shown in blue and red) can be cleaved by NcoI, BclI, SalI, BamHI, EcoRI, and BglII. The NcoI and BamHI sites of this MCS were used to clone an NcoI-BamHI DNA fragment (1924 bp) containing an ORF coding the firefly luciferase, which was purified from a pGL3 plasmid (P_Luc). pMos1 and pHsmar1 DNA fragments were synthesized by ATG:biosynthetics GMBh (Merzhausen, Germany) and each cloned in a pUC19 plasmid. (DOCX) [file pgen.1005902.s003.docx]

**pMos1.**

**TA**CCAGGTGTACAAGTAGGGAATGTCGGTTCGAACATATAGATGTCTCGCAAACGTAAAT

ATTTATCGATTGTCATAAAACTTTGACCTTGTGAAGTGTCAACCTTGACTGTCGAACCAC

CATAGTTTGGCGCAAATTGAGCGTCATAATTGTTTACTCTCAGTGCAGTCAACCCATGGT

GATCAGTCGACGGATCCGAATTCAGATCT

**pHsmar1.**

**TA**Ttaggttggtgcaaaagtaattgcggtttttgcattgttggaatttgccgtttgatat

tggaatacattcttaaataaatgtggttatgttatacatcattttaatgcgcatttctcg

ctttacgtttttttgctaatgacttattacttgctgtttattttatgtttattttagact

CCATGGTGATCAGTCGACGGATCCGAATTCAGATCT
