# Supplementary material for: Mariner Transposons Contain a Silencer: Possible Role of the Polycomb Repressive Complex 2
Source: PLoS Genet. 2016 Mar 3;12(3):e1005902. doi: 10.1371/journal.pgen.1005902 (PMC4777549; doi:10.1371/journal.pgen.1005902)
Supplement: S4 Fig — (a) Location of the Δ81 to Δ85 MOS1 DNA segments within the sequence of the Δ8-MOS1 segment. Δ8, black; Δ81, red; Δ82, blue; Δ83, green; Δ84, purple; Δ85, orange. The region absent in the short version of Δ81 (short Δ81 probe in Fig 7F) is highlighted in grey in the sequence of Δ81. Transcription factor binding sites frequently found within PREs in drosophila are shown and are highlighted in blue for YY1 or Pho, green for Ezh2 or Zeste, turquoise and pink for the GAGA and GTGT factors, respectively. Other binding sites are in red for NRSF, grey for NFAT-5, and black for Alx1. (b) Comparison of effects of Δ7-MOS1 and Δ8-MOS1 on the expression of the Firefly and the Renilla luciferase marker genes using transient expression assays in HeLa cells. The assays were performed with Δ7 and Δ8 segments cloned in + orientation. Each histogram bar corresponds to the median value obtained from three experiments done in triplicate. Bars corresponded to quartiles 1 and 3. The median ratios RLU from Firefly/RLU from Renilla were calculated as indicated in Fig 2. The area where the ratios “RLU from Firefly/RLU from Renilla” were above 1 (i.e. where no strong silencer effect is observed) is coloured in grey. No significant effect was found between both plasmid constructs (p<0.05). (DOCX) [file pgen.1005902.s004.docx]

**a.**

1/1 31/11 61/21 91/31

| | | |

GGTCATTTTTCTCCATGACAACGCTCCATCACATACGGCAAGAGCGGTTCGCGACACGTTGGAAACACTCAATTGGGAAGTGCTTCCGCATGCGGCTTACTCACCAGACCTGGCCCCATC

V I F L H D N A P S H T A R A V R D T L E T L N W E V L P H A A Y S P D L A P S

GGTCATTTTTCTCCATGACAACGCTCCATCACATACGGCAAGAGCGGTTCGCGACACGTTGGAAACACTCAATTGGGAAGTGCTTCCGCATGCGGCTTAC

CGCGACACGTTGGAAACACTCAATTGGGAAGTGCTTCCGCATGCGGCTTACTCACCAGACCTGGCCCCATC

TCACCAGACCTGGCCCCATC

121/41 151/51 181/61 211/71

| | | |

CGATTACCACCTATTCGCTTCGATGGGACACGCACTCGCTGAGCAGCGCTTCGATTCTTACGAAAGTGTGAAAAAATGGCTCGATGAATGGTTCGCCGCAAAAGACGATGAGTTCTACTG

D Y H L F A S M G H A L A E Q R F D S Y E S V K K W L D E W F A A K D D E F Y W

CGATTACCACCTATTCGCTTCGATGGGACAC

CGATTACCACCTATTCGCTTCGATGGGACACGCACTCGCTGAGCAGCGCTTCGATTCTTACGAAAGTGTGAAAAAATGGCTC

GCACTCGCTGAGCAGCGCTTCGATTCTTACGAAAGTGTGAAAAAATGGCTCGATGAATGGTTCGCCGCAAAAGACGATGAGTTCTACTG

GATGAATGGTTCGCCGCAAAAGACGATGAGTTCTACTG

241/81 271/91 301/101

| | |

GCGTGGAATCCACAAATTGCCCGAGAGATGGGAAAAATGTGTAGCTAGCGACGGCAAATACTTTGAATAA

R G I H K L P E R W E K C V A S D G K Y F E *

GCGTGGAATC

GCGTGGAATCCACAAATTGCCCGAGAGATGGGAAAAATGTGTAGCTAGCGACGGCAAATACTTTGAATAA

**b.**
