# Supplementary material for: Mariner Transposons Contain a Silencer: Possible Role of the Polycomb Repressive Complex 2
Source: PLoS Genet. 2016 Mar 3;12(3):e1005902. doi: 10.1371/journal.pgen.1005902 (PMC4777549; doi:10.1371/journal.pgen.1005902)
Supplement: S5 Fig — Black: Δ8 mariner segments; black+grey: Δ7 mariner segments. Transcription factor binding sites frequently found within PREs in drosophila are shown in blue for YY1, in green for Zeste, in turquoise for the GAGA factor and in pink for the GTGT factors. Arrows above the nucleic acid sequences indicate the orientation of each motif. NRSF binding sites are highlighted in red and typed in white. With respect to NRSF binding sites that are conserved in position in all MLEs, the other motifs are arranged in different configurations, ordering and spacing in each element. This indicated that these motifs would not have been conserved in orthologous positions across MLEs during their evolution. Putative NFAT-5 binding sites are highlighted in boxes. (DOCX) [file pgen.1005902.s005.docx]

***Himar1***

-----> <---------

TCGGCTGAGTTGACAGCGACCGGTGAACCGTCTCCGAAGCGTGGAAAGACTCAAAAGTCCGCTGGCAAAGTAATGGCCTCTGTTTTTTGGGATGCGCATG

<---------

GAATAATTTTTATCGATTATCTTGAGAAGGGAAAAACCATCAACAGTGACTATTATATGGCGTTATTGGAGCGTTTGAAGGTCGAAATCGCGGCAAAACG

GCCCCACATGAAGAAGAAAAAAGTGTTGTTCCACCAAGACAACGCACCGTGCCACAAGTCATTGAGAACGATGGCAAAAATTCATGAATTGGGCTTCGAA

---------------------------> <-----

TTGCTTCCCCACCCGCCGTATTCTCCAGATCTGGCCCCCAGCGACTTTTTCTTGTTCTCAGACCTCAAAAGGATGCTCGCAGGGAAAAAATTTGGCTGCA

<---------

ATGAAGAGGTGATCGCCGAAACTGAGGCCTATTTTGAGGCAAAACCGAAGGAGTACTACCAAAATGGTATCAAAAAATTGGAAGGTCGTTATAATCGTTG

TATCGCTCTTGAAGGGAACTATGTTGAAGGATCC

***Mcmar1***

<--------- ----->

AAGCAATGGCTTGCAGTTGGGCAAGATGCTAATGCAACTCCAAAGCAGCTTCATCCAAAGAAACAGCTATTGAGTGTTTGGTGGTGCGTTCATGGAATTG

TCTACTGGGAGCTTCTTCCTTTAAATCGCACTATAACATCAGAGGTTTACTGTGAGCAACTACATCGTGTACAACAACAACTACGTCGTCCTCCATATAC

<----- ---->

GGTTTGGGCGAGAAAGGGCATACTATTCCAACAAGACGGAGCTCGTCCACATGTGTCTGCTGTAACACGAAAGAAGATAGAAGATCTTGGATGGGATATT

<----

--------------------------->

CTTGAACATAGTCCTTACTCTCCAGATCTAGCACCGTCAGACTATTATTTGTTTAGTCCTCTGAAAGATTTTCTACGTGGAAAACAATTTTCAAATGAGG

<----- <---------

AAGAAATTTGCACAGCACTGAAGAATTTTTTTGACTCAAAAGGGCCTGAATGGTATCGCAAAGGGATTGAAAAGCTTCCTAACCTTTGGGAACGATGCAT

TCAATGTAATGGAATTATTTCTATGAAGGATCC

***Hsmar1***

<----GCTCAGTGGTTGGACCGAGAAGAAGCTCCAAAGCACTTCCCAAAGCCAAACTTGCACCAAAAAAAGGTCATGGTCACTGTTTGGTGGTCTGCTGCCGGTC

<-----

TGATCCACTACAGCTTTCTGAATCCCGGCGAAACCATTACATCTGAGAAGTATGCTCAGCAAATCGATGAGATGCACCGAAAACTGCAACGCCTGCAGCC

GGCATTGGTCAACAGAAAGGGCCCAATTCTTCTCCACGACAACGCCCGACCGCACGTCGCACAACCAACGCTTCAAAAGTTGAACGAATTGGGCTACGAA

---------> <------------------------------->

GTTTTGCCTCATCCGCCATATTCACCTGACCTCTCGCCAACCGACTACCACTTCTTCAAGCATCTCGACAACTTTTTGCAGGGAAAACGCTTCCACAACC

AGCAGGATGCAGAAAATGCTTTCCAAGAGTTCGTCGAATCCCGAAGCACGGATTTTTACGCTACAGGAATAAACAAACTTATTTCTCGTTGGCAAAAATG

<---------

TGTTGATTGTAATGGTTCCTATTTTGATTAA

***Hsmar2***

<--------- <-----

AAGCAATGGCTACCAAGAGGTGGAAGTGGTCCAGTCAAAGCAAAAGCGGACTGGTCAAGAGCAAAGGTCATGGCAACAGTTTTTTGGGATGCTCAAGGCA

---->

TTTTGCTTGTTGACTTTCTGGAGGGCCAAAGAACGATAACATCTGCTTATTATGAGAGTGTTTTGAGAAAGTTAGCCAAAGCTTTAGCAGAAAAACGCCC

------> <----- <---- ---->

GGGAAAGCTTCACCAGAGAGTCCTTCTCCACCACGACAATGCTCCTGCTCATTCCTCTCATCAAACAAGGGCAATTTTGCGAGAGTTTCGATGGGAAATC

---------------------------> --------->

ATTAGGCATCCACCTTACAGTCCTGATTTGGCTCCTTCTGACTTCTTTTTGTTTCCTAATCTTAAAAAATCTTTAAAGGGCACCCATTTTTCTTCAGTTA

<--------- <---------

ATAATGTAAAAAAGACTGCATTGACATGGTTAAATTCCCAGGACCCTCAGTTCTTTAGGGATGGACTAAATGGCTGGTATCATCGCTTACAAAAGTGTCT

TGAACTTGATGGAGCTTATGTTGAGAAATAA
