# Supplementary material for: Mariner Transposons Contain a Silencer: Possible Role of the Polycomb Repressive Complex 2
Source: PLoS Genet. 2016 Mar 3;12(3):e1005902. doi: 10.1371/journal.pgen.1005902 (PMC4777549; doi:10.1371/journal.pgen.1005902)
Supplement: S6 Fig — Conserved motifs were searched within the nucleic acid sequences corresponding to the Δ7 DNA segment of 34 mariner elements using the MEME facilities at http://meme.sdsc.edu/meme/cgi-bin/meme.cgi. A single conserved motif of 50 nucleotides was found and was located in the region encoding one of the two highly conserved peptide motifs in the mariner transposase, PHxxYSPDLAPxD [34]. We found that this conserved 50 bp motif also contained a 29 bp NRSE with a 10 bp spacer between both conserved moieties, rather than a 2 bp spacer as found in the cardinal NRSF binding site (RE1). Charlatan is the ortholog of NRSF in diptera. The names of each of the 34 mariner elements are shown on the right side of the figure. Their names, accession numbers and host species for each element are: Tvmar1, AY282463 Trichomonas vaginalis; Ahmar1, AB056896 Adoxophyes honmai; Armar1, AB056894 Ascogaster reticulatus; Cpmar1, U11641 Chrysoperla plorabunda; Mpmar1, U11649 Mantispa pulchella; Damar1, U11656 Drosophila ananassae; Himar1, U11646 Haematobia irritans; Hsmar2, U49974 Homo sapiens; Bytmar1, AJ507226 Bythogrea thermydron; Acmar1, AB081476 Apis cerena; Ammar1, U19902 for Apis mellifera; Ccmar1, U40493 Ceratitis capitata; Demar1, U08094 Drosophila erecta; Famar1, AY226507 Forficula auricularia; Gpmar1, U18308 Glossina palpalis; Aamar1, AB006464 Attacus atlas; Dtmar1, X79719 Dugesia tigrina; Funmar1, AB055188 Fungia sp. Kusabiraishi; Hbmar1, U04455 Heterorhabditis bacteriophaga; Hcmar1, M63844 Hyalophora cecropia; Hsmar1, U52077 Homo sapiens; Cemar1, M98552 Caenorhabditis elegans; Cemar2, X77804 C. elegans; Mcmar1, H20772 Meloidogyne chitwoodi; Bcmar1, AF349133 Bactrocera tryoni; Botmar1, consensus sequence (personnal data) Bombus terrestris; Dsecmar1, AF035569 Drosophila sechellia; Madmar1, U24436 Mayetiola destructor; Mbmar1, AF465247 Mamestra brassicae; Mlmar1, AC182003 (element from 70120 to 71051) Myotis lucifugus; Dmmar1 = Mos1, X78906 Drosophila mauritiana; Momar1, U12279 Metaseuili [file pgen.1005902.s006.docx]

P H x x Y S P D L A P x D

Tvmar1 AAAGTTGTTCCAAACCCAATTTATTCACCTGATATTGCACCCTCTGACTT

Ahmar1 GAATTGCTTCCTCATCCACCGTATTCTCCAGATCTGGCCCCCAGCGACTA

Armar1 GAATTGCTTCCTCATCCACCGTATTCTCCAGATCTGGCCCCCAGCGACTA

Cpmar1 GAATTGCTTCCCCACCCGCCGTATTCTCCAGATCTGGCCCCCAGCGACTT

Mpmar1 GAATTGCTTCCGCATCCACCGTATTCTCCAGATTTGGCCCCCAGCGACTA

Damar1 GAATTGCTTCCCCACCCACCGTATTCTCCAGATCTGGCCCCCAGCGACTT

Himar1 GAATTGCTTCCCCACCCACCGTATTCTCCAGATCTGGCCCCCAGCGACTT

Hsmar2 GAAATCATTAGGCATCCACCTTACAGTCCTGATTTGGCTCCTTCTGACTT

Bytmar1 GAGCAGCTCAATCACCCACCCTACAGTCCGGACCTGGCTCCCAGCGACTA

Acmar1 GAAGTGCTACCACATCCACCATATTCGCCCGATCTGGCACCTTCAGATTA

Ammar1 GATGTTTTGCCACATTCACTATATAGTCCTGACCTTGCACCATCTGATTA

Ccmar1 GAAATCATGCCGCATTCCCCATATTCACCCGACATTGCACCTTCTGATTA

Demar1 GAGGTTTTATCGCATCCACCATATAGCCCGGACATAGCGCCAAGTGATTA

Famar1 GATGTTTTGCCACATCCACCATATAGTCCTGACCTTGCACCATCTGATTA

Gpmar1 GAAGTTTTAATGCATCCGCCATACAGTCCGGACTTGGCACCAAGCGATTA

Aamar1 GAATGTCTAAGACATCCACCGTATTCCCCGGACCTTGCTCCAACTGATTA

Dtmar1 GAAACTCTTCGCCACCCAACATATTCACCGGATCTCGCACCCACGGACTG

Funmar1 GAATGTCTAAGACATCCACCGTATTCCCCGGATCTTGCTCCAACTGATTA

Hbmar1 GAGACTCTACCTTACCCAGTTTACTCACCAGACCTCTCTTCTACCAATTA

Hcmar1 GAATGTCTGCGACATCCACCGTACTCCCCGGACCTTGCTCCAATAGATTA

Hsmar1 GAAGTTTTTCCTCATCCGCCATATTCACCTGACCTCTCGCCAACCGACTA

Cemar1 ACTGTTTTACCGCATCCACCATATTCTCCAGATCTTGCACCAACCGACTA

Cemar2 CAAATTTTGTCTTACCCATCGTATTCGCCGGGCTTGGCTCCTACTGACTA

Mcmar1 GATATTCTTGAACATAGTCCTTACTCTCCAGATCTAGCACCGTCAGACTA

Bcmar1 GGGGTTTTGATGCATCCACCATATAGCCCTGACCGTGCACCATCGGACTA

Botmar1 GAAATACCTTCGCACGCGGCTTACTCGCCAGACTTGGCTCCGTCCGATTA

Dsecmar1 GAAGTGCTTCCGCATGCGGCTTACTCACCAGACCTGGCCCCGTCCGATTA

Madmar1 GAAGTTTTACCCCACCCGCCTTATAGCCCAGACCTTGCCCCTTCTGACTA

Mbmar1 GAAGTGCTTCCGCATGCGGCTTACTCACCAGACCTGGCCCCATCCGATTA

Mlmar1 GAAGTATTAACCCACCCGCCGTATTCACCAGACCTTGCTCCTTCAGATTA

mos1 GAAGTGCTTCCGCATGCGGCTTACTCACCAGACCTGGCCCCATCCGATTA

Momar1 GAAGTTCTGACACACCCTCGCTATTCTCCAGACTTGGCCCCCTCAGATTA

Mudmar1 GAAGTGCTTCCGCATGCGGCTTACTCACCAGACCTGGCCCCATCCGATTA

Sinvmar1 GAAGTTCTGCCGCACCCAGCCTACTCTTCAGACTTGGCGTCGGATTACCA

RE1 Consensus ttcagcacc........acggacagcgcc

Charlatan consensus bbhAsmvmm.........CnGACvknnCC
