# Supplementary material for: Mariner Transposons Contain a Silencer: Possible Role of the Polycomb Repressive Complex 2
Source: PLoS Genet. 2016 Mar 3;12(3):e1005902. doi: 10.1371/journal.pgen.1005902 (PMC4777549; doi:10.1371/journal.pgen.1005902)
Supplement: S7 Fig — (a) The RE1 probe corresponds to the RE1 binding site located upstream of the vesicular acethylcholine (VAChT) transporter and choline acethyltransferase (ChAT) genes in the human cholinergic gene locus. A 120 bp DNA segment was amplified by PCR from the pKL-7 plasmid [38]. Primers are highlighted in grey and the RE1 site in yellow. ATTO fluorochrome located at the outer 3’ end in the labelled probe is shown in bold. (b) Set-up of the EMSA for chn. Lane 1 corresponds to a probe control. Lanes 2 to 4 were obtained with a nuclear extract (NE) containing chn. Because chn was not tagged with a peptide and we did not have anti-chn antibodies, the specificity of the shifted complex observed in lane 2 was verified in lanes 3 and 4 by observing binding with a specific DNA binding competitor (CBE-1: 5’-GGCCGTTCAGCACCACCGCCATTGGTCGCGC-3’ [37]) and a non-specific competitor (MCS; sequence in S8 Fig), as described previously [37–38]. (c) Set-up of the EMSA for HsNRSF and fuguNRSF. Lanes 1 and 10 correspond to probe controls. Lanes 2 through 5 and 11 through 14 are negative controls obtained with an NE prepared from HeLa cells transfected with an empty expression plasmid: pCS2+. Lanes 6 through 9 were obtained with an NE containing HsNRSF fused to a FLAG tag. The specificity of the shifted complex observed in lane 6 was verified in lane 8 in which the anti-FLAG monoclonal antibody (Anti-FLAG MAb) creates a super-shifted complex (red stars). Lanes 15 to 18 were obtained with an NE containing fuguNRSF fused to a myc tag. The specificity of the shifted complex observed in lane 15 was verified in lane 16 in which the anti-myc monoclonal antibody (Anti-myc MAb) creates a super-shifted complex. Lanes 7, 9, 17 and 18 were negative controls that support the conclusion that the super-shifted complexes observed in lanes 8 and 16 were not due to a non-specific effect of the MAb. Complexes were separated on a 6% PAGE in 0.25X TBE gel. Together, these results support that the three NRSF syst [file pgen.1005902.s007.docx]

**a.** RE1: length = 120 bp; PM = 79200 Da, i.e. 1 ng = 12,62 10^-15^ mole.

aagtgaagaggaagccagaggggcggggcctccgtgggaggcttcttggttgcggtggggccggggccagcaccctggacagctcccggcggctggcggggagaagaggctttgccaggt**-ATTO**

**b.**

**c.**

**
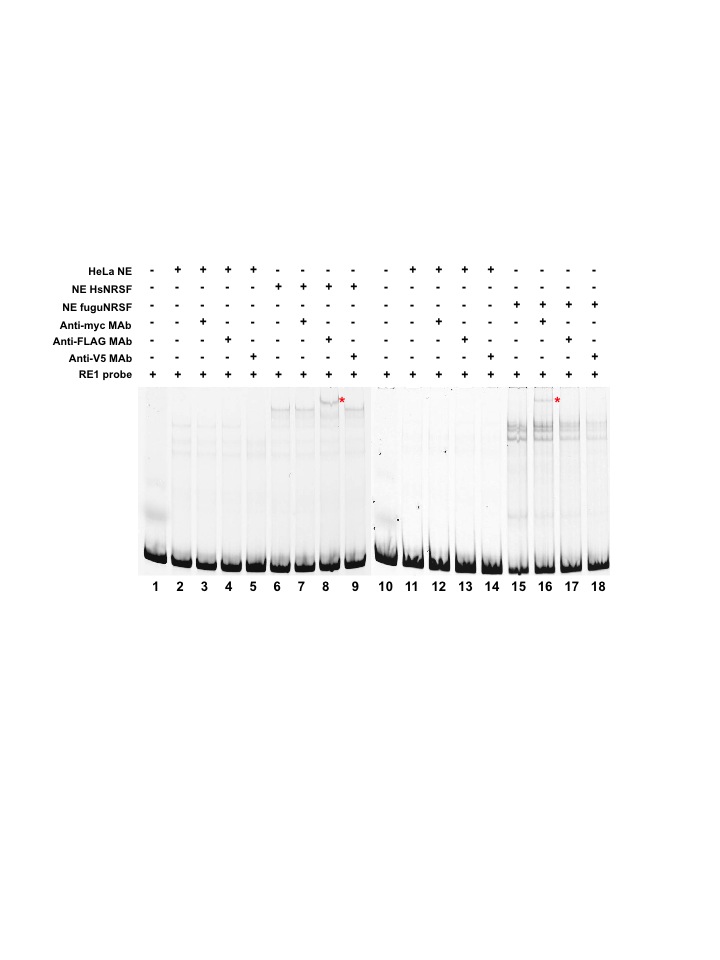
**
