# Supplementary material for: Mariner Transposons Contain a Silencer: Possible Role of the Polycomb Repressive Complex 2
Source: PLoS Genet. 2016 Mar 3;12(3):e1005902. doi: 10.1371/journal.pgen.1005902 (PMC4777549; doi:10.1371/journal.pgen.1005902)
Supplement: S8 Fig — Location of the variant segments within the sequences of the Δ8-MOS1 (a) and Δ8-HSMAR1 (b). Names of each variant are indicated at the 3’ end of its sequence. Dashes indicated positions that are present in each fragment. Conserved DNA binding motifs described in Fig 6B are highlighted in red for NRSF, blue for YY1 or Pho, green for Ezh2 or Zeste, turquoise and pink for the GAGA and GTGT factors, grey for NFAT-5, and black for Alx1, respectively. (c) Expression of the Firefly and the Renilla luciferase marker genes using transient expression assays in HeLa cells. The assays were performed with Δ8-HSMAR1 and five variants cloned in + orientation. Each bar corresponds to the median value obtained from three experiments done in triplicate. Bars corresponded to quartiles 1 and 3. The median ratios RLU from Firefly/RLU from Renilla were calculated as indicated in Fig 2. The area where the ratios “RLU from Firefly/RLU from Renilla” were above 1 (i.e. where no strong silencer effect is observed) is coloured in grey. * indicates a significant difference (p<0.05) with the P_Luc controls. ** indicates a significant difference (p<0.05) with HS2_P_Luc_Δ8-HSMAR1. (DOCX) [file pgen.1005902.s008.docx]

**a.**

1/1 31/11 61/21 91/31

| | | |

GGTCATTTTTCTCCATGACAACGCTCCATCACATACGGCAAGAGCGGTTCGCGACACGTTGGAAACACTCAATTGGGAAGTGCTTCCGCATGCGGCTTACTCACCAGACCTGGCCCCATC

-------------------------------------------------------------------------------------- -----

--------------------------------------------------------------------------------------ATTAGCGCCGTGGAGTGCCGCGGTGAGAC-----

121/41 151/51 181/61 211/71

| | | |

CGATTACCACCTATTCGCTTCGATGGGACACGCACTCGCTGAGCAGCGCTTCGATTCTTACGAAAGTGTGAAAAAATGGCTCGATGAATGGTTCGCCGCAAAAGACGATGAGTTCTACTG

------------------------------------------------------------------------------------------------------------------------

------------------------------------------------------------------------------------------------------------------------

241/81 271/91 301/101

| | |

GCGTGGAATCCACAAATTGCCCGAGAGATGGGAAAAATGTGTAGCTAGCGACGGCAAATACTTTGAATAA Δ8MOS1

---------------------------------------------------------------------- Δ8MOS1-ΔNRSF

---------------------------------------------------------------------- Δ8MOS1-^mut^NRSF

**b.**

1/1 31/11 61/21 91/31

| | | |

CCCAATTCTTCTCCACGACAACGCCCGACCGCACGTCGCACAACCAACGCTTCAAAAGTTGAACGAATTGGGCTACGAAGTTTTTCCTCATCCGCCATATTCACCTGACCTCTCGCCAAC

-------------------------------------------------------------------------------------- -----

--------------------------------------------------------------------------------------TTCAGTTCCCACGCCTATCACACACGACC-----

----------------------------------

-----

------------------------- -----

------ -----

121/41 151/51 181/61 211/71

| | | |

CGACTACCACTTCTTCAAGCATCTCGACAACTTTTTGCAGGGAAAACGCTTCCACAACCAGCAGGATGCAGAAAATGCTTTCCAAGAGTTCGTCGAATCCCGAAGCACGGATTTTTACGC

------------------------------------------------------------------------------------------------------------------------

------------------------------------------------------------------------------------------------------------------------

------------------------------------------------------------------------------------------------------------------------

------------------------------------------------------------------------------------------------------------------------

------------------------------------------------------------------------------------------------------------------------

------------------------------------------------------------------------------------------------------------------------

241/81 271/91 301/101

| | |

TACAGGAATAAACAAACTTATTTCTCGTTGGCAAAAATGTGTTGATTGTAATGGTTCCTATTTTGATTAA Δ8Hsmar1

---------------------------------------------------------------------- Δ8Hsmar1-ΔNRSF

---------------------------------------------------------------------- Δ8Hsmar1-^mut^NRSF

---------------------------------------------------------------------- Δ8Hsmar1-[86-310]

---------------------------------------------------------------------- Δ8Hsmar1-[115-310]

---------------------------------------------------------------------- Δ8Hsmar1-61-310]-ΔNRSF

--------------------------------------------------------------- Δ8Hsmar1-[81-310]-ΔNRSF

**c.**
