# Supplementary material for: Mariner Transposons Contain a Silencer: Possible Role of the Polycomb Repressive Complex 2
Source: PLoS Genet. 2016 Mar 3;12(3):e1005902. doi: 10.1371/journal.pgen.1005902 (PMC4777549; doi:10.1371/journal.pgen.1005902)
Supplement: S11 Fig — (a) mariner elements and Δ8 mariner segments were counted using the RepeatMasker annotation in the Hg19 version. (*) number of elements with a maximum of 40 bp truncated at their 5’ and-or 3’ ends. Damaged Δ8 mariner segments are DNA regions containing one or several DNA insertions or deletions. (b) Conservation profiles in terms of sequence divergence and integrity of Hsmar1 and Hsmar2 elements in the human genome. The horizontal axes are calibrated to the size of the mariner elements. The vertical axis represents the number of stacked mariner elements. Each horizontal lane corresponds to one mariner element that consists in 1 to 6 high-scoring segment pairs (HSP). For Hsmar1, 469 elements are in 1 HSP, 106 in 2 HSPs, 16 in 3 HSPs, and 1 in 1 HSP. For Hsmar2, 838 elements are in 1 HSP, 280 in 2 HSPs, 6, in 3 HSPs, 28 in 1 HSP, 8 in 5 HSPs, and 3 in 6 HSPs. Mariner elements were ranked in the stacking from the best RepeatMasker scores to the worst. In the right margin a coloured scale from red to blue is shown, representing the sequence divergence of each element with its reference element (Hsmar1, AccN° HSU52077; Hsmar2; Acc N° HSU49974). Black bars above the horizontal axis show the location of the Δ8 segments. (DOCX) [file pgen.1005902.s011.docx]

**a.**

| Number of loci | *Hsmar1* | *Hsmar2* |
| --- | --- | --- |
| in which is inserted a *mariner* element | 592 | 1240 |
| with a full length *mariner* element | 165 + 196^*^ | 268 + 327^*^ |
|  |  |  |
| containing a Δ8 *mariner* segment (Sil+) | 315 | 644 |
| with no Δ8 *mariner* segment (Sil-) | 236 | 485 |
| containing a damaged Δ8 *mariner* segment (U) | 24 | 111 |
|  |  |  |
| Sil+ with a location into a genic region | 95 | 240 |
| Sil+ with a location into an intergenic region | 220 | 404 |
| Sil - with a location into a genic region | 154 | 291 |
| Sil - with a location into an intergenic region | 82 | 194 |

**b.**


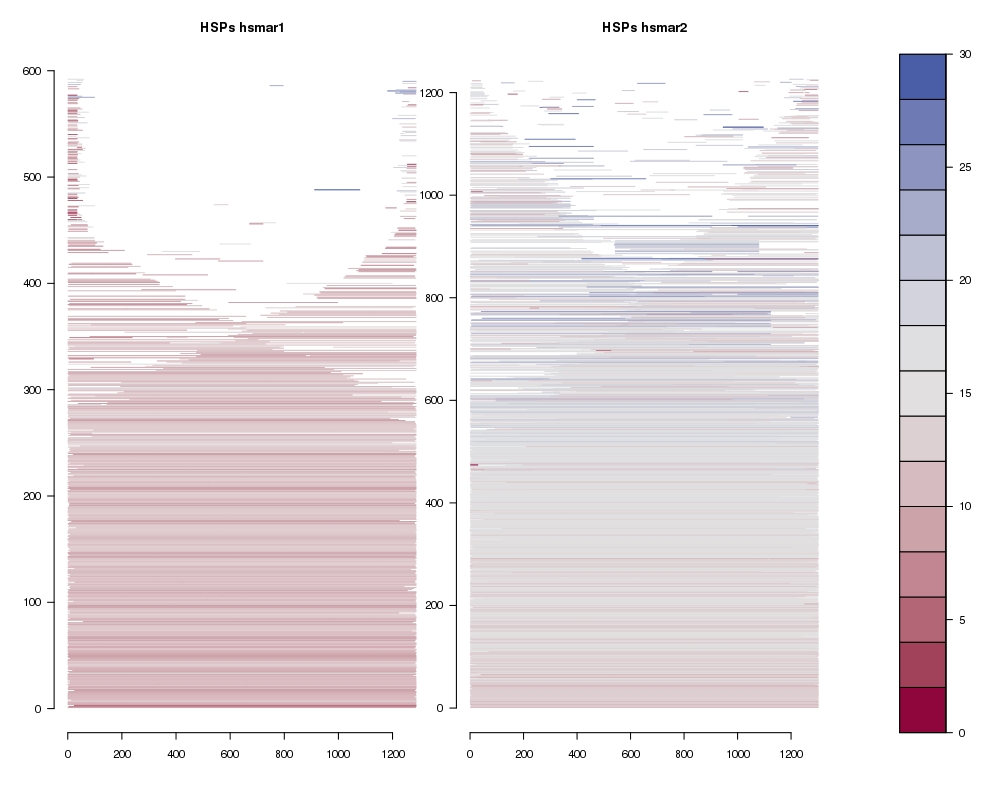

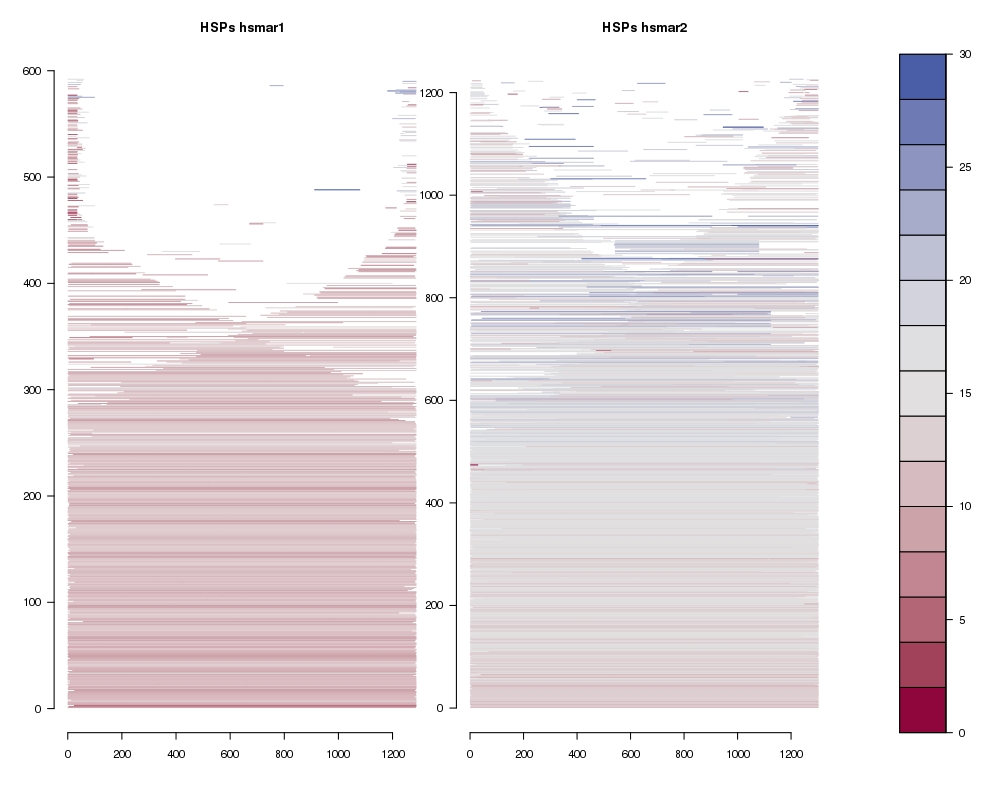


***Hsmar1 Hsmar2***
