# Supplementary material for: Mariner Transposons Contain a Silencer: Possible Role of the Polycomb Repressive Complex 2
Source: PLoS Genet. 2016 Mar 3;12(3):e1005902. doi: 10.1371/journal.pgen.1005902 (PMC4777549; doi:10.1371/journal.pgen.1005902)
Supplement: S3 Table — (DOCX) [file pgen.1005902.s016.docx]

**Table S3. Chromatin status of the 8 *Hsmar1* Δ7 silencers in 14 human cell lines**

| **Cell lines** | **N° of the *Hsmar1* Δ7 silencer** | | | | | | | | **Consensual status for each cell type** |
| --- | --- | --- | --- | --- | --- | --- | --- | --- | --- |
|  | **1** | **2** | **3** | **5** | **6** | **7** | **8** | **9** |  |
| Dnd1 | P | T | P | P | ucs | P | H | P/H | P |
| GM12878 | ucs | P/H | ucs | P | ucs | ucs | H | H | H |
| H1.hESC | ucs | ucs | ucs | ucs | ucs | P | H | T | ucs |
| HeLa.S3 | P | ucs | ucs | T | ucs | P | H | P | P |
| HepG2 | ucs | ucs | H | P | ucs | P | H | P | P |
| HMEC | H | ucs | ucs | ucs | ucs | ucs | ucs | H | ucs |
| HSMM | H | P | ucs | H | ucs | P | P | H | P/H |
| HSMMtube | H | P | ucs | P | ucs | P | ucs | P | P |
| HUVEC | H | ucs | ucs | ucs | H | P | H | P/H | H |
| K562 | ucs | H | H | P | H | P/T/H | H | P | H |
| NH.A | H | ucs | P | T | ucs | ucs | ucs | H | H |
| NHFD.ad | H | ucs | ucs | ucs | ucs | P | H | H | H |
| NHEK | H | ucs | ucs | P | H | ucs | ucs | P/H | H |
| NHLF | H | ucs | ucs | H | H | ucs | H | H | H |
| **Consensual status for each locus** | H | P | P/H | P | H | P | H | H |  |

P, T, and H indicated polycomb, trithorax and Su(var)39/HP1 status, respectively. “ucs“ indicated an absence of co-localized peaks that was considered under our analysis conditions as an undetermined chromatin status. When a mixed status was found it was indicated here by P/T/H and P/H. The consensual status for each cell type (right column) or each locus (bottom lane) was determined using a majority rule of 50% on cases having an annotation. Column highlighted in grey indicated *Hsmar1* Δ7 silencers located in genic regions.
