# Supplementary material for: Mariner Transposons Contain a Silencer: Possible Role of the Polycomb Repressive Complex 2
Source: PLoS Genet. 2016 Mar 3;12(3):e1005902. doi: 10.1371/journal.pgen.1005902 (PMC4777549; doi:10.1371/journal.pgen.1005902)
Supplement: Supplementary file 12 [file pgen.1005902.s002.docx]

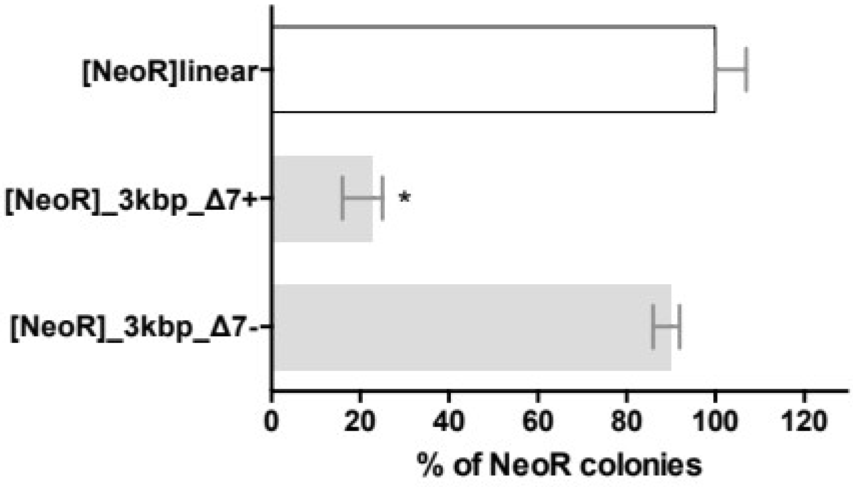
[NeoR]linear
[NeoR]_3kbp_Δ7+
*
[NeoR]_3kbp_Δ7-
0
20
40
60
80
100
120
% of NeoR colonies
