# Supplementary material for: Mariner Transposons Contain a Silencer: Possible Role of the Polycomb Repressive Complex 2
Source: PLoS Genet. 2016 Mar 3;12(3):e1005902. doi: 10.1371/journal.pgen.1005902 (PMC4777549; doi:10.1371/journal.pgen.1005902)
Supplement: Supplementary file 13 [file pgen.1005902.s009.docx]

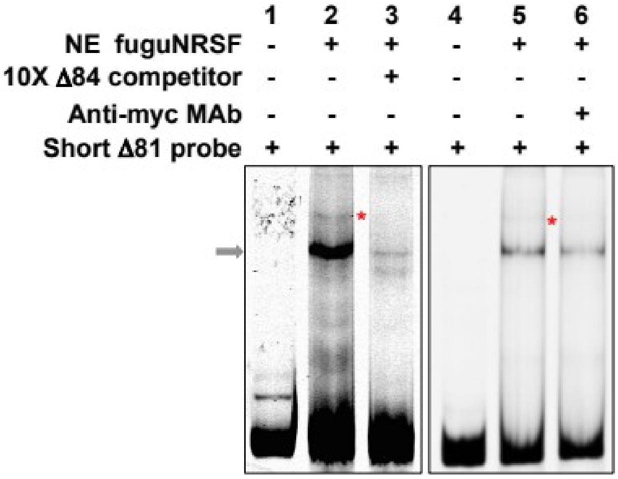
1 2 3 4 5 6
NE fuguNRSF - + + - + +
10X Δ84 competitor - - + - - -
Anti-myc MAb - - - - - +
Short Δ81 probe + + + + + +
*
*
