# Supplementary material for: Mariner Transposons Contain a Silencer: Possible Role of the Polycomb Repressive Complex 2
Source: PLoS Genet. 2016 Mar 3;12(3):e1005902. doi: 10.1371/journal.pgen.1005902 (PMC4777549; doi:10.1371/journal.pgen.1005902)
Supplement: Supplementary file 14 [file pgen.1005902.s010.docx]

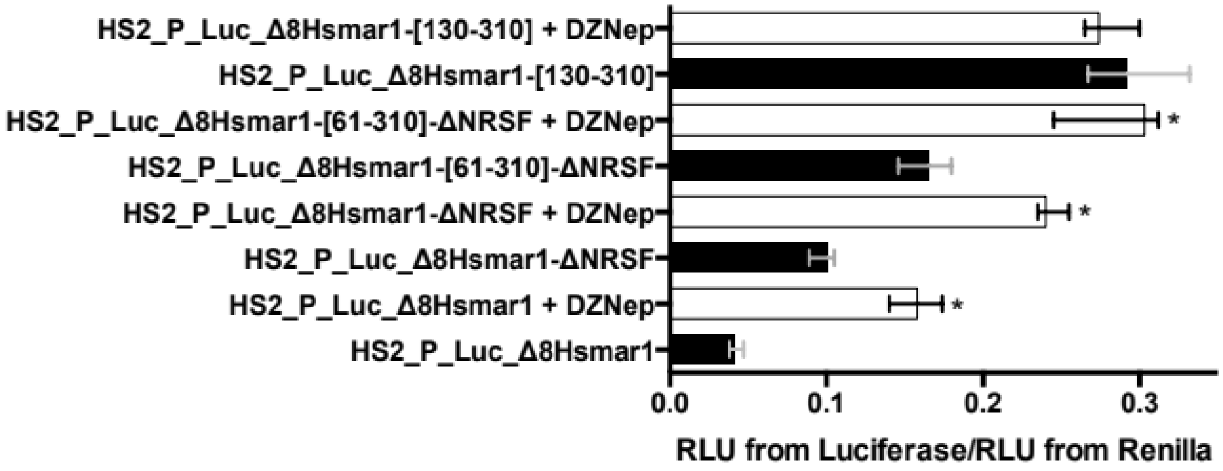
HS2_P_Luc_Δ8Hsmar1-[130-310] + DZNep
HS2_P_Luc_Δ8Hsmar1-[130-310]
HS2_P_Luc_Δ8Hsmar1-[61-310]-ΔNRSF + DZNep
HS2_P_Luc_Δ8Hsmar1-[61-310]-ΔNRSF
HS2_P_Luc_Δ8Hsmar1-ΔNRSF + DZNep
HS2_P_Luc_Δ8Hsmar1-ΔNRSF
HS2_P_Luc_Δ8Hsmar1 + DZNep
HS2_P_Luc_Δ8Hsmar1
*
*
*
0.0
0.1
0.2
0.3
RLU from Luciferase/RLU from Renilla
